# Polyinosinic/Polycytidylic Lipid Nanoparticles Enhance Immune Cell Infiltration and Improve Survival in the Glioblastoma Mouse Model

**DOI:** 10.1021/acs.molpharmaceut.4c00875

**Published:** 2024-11-18

**Authors:** Melanie M. T. Brüßeler, Alaa Zam, Víctor M. Moreno-Zafra, Nadia Rouatbi, Osama W. M. Hassuneh, Alessia Marrocu, Revadee Liam-Or, Hend Mohamed Abdel-Bar, Adam Alexander Walters, Khuloud T. Al-Jamal

**Affiliations:** † Institute of Pharmaceutical Science, King’s College London, Franklin-Wilkins Building 150 Stamford Street, London SE1 9NH, U.K.; ‡ Ludwig Maximilians University, Bayern, Munich, München 80539, Germany; § Department of Pharmacology and Pharmacy, Li Ka Shing Faculty of Medicine, The University of Hong Kong, Hong Kong Special Administrative Region, Hong Kong 999077, China; ∥ Department of Pharmaceutics, Faculty of Pharmacy, University of Sadat City, P.O. Box 32958, El Sadat, Egypt

**Keywords:** doxorubicin, pIpC, CpG, glioblastoma, lipid nanoparticle

## Abstract

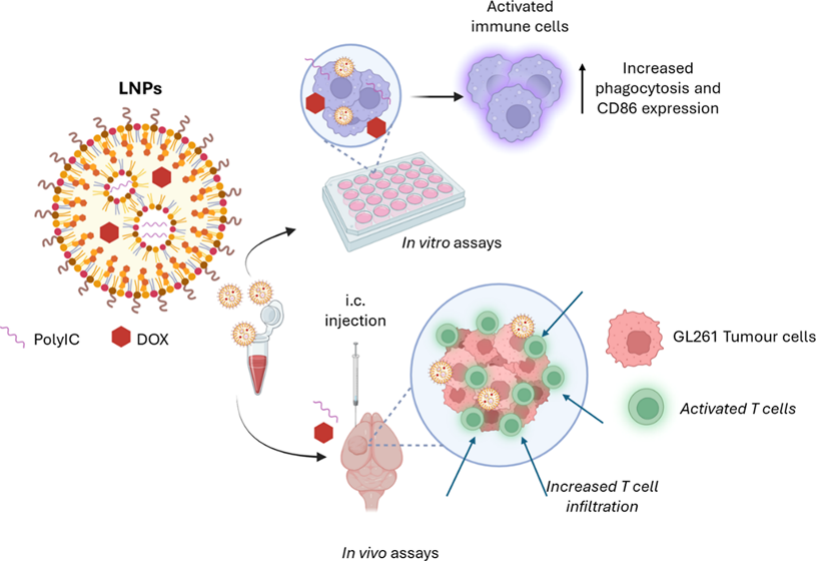

Glioblastoma (GBM) immunotherapy is particularly challenging due to the pro-tumorigenic microenvironment, marked by low levels and inactive immune cells. Toll-like receptor (TLR) agonists have emerged as potent immune adjuvants but failed to show improved outcomes in clinical trials when administered as a monotherapy. We hypothesize that a combined nanoparticulate formulation of TLR agonist and immunogenic cell death-inducing drug (doxorubicin) will synergize to induce improved GBM immunotherapy. Lipid nanoparticle (LNP) formulations of the TLR agonists CpG and polyinosinic/polycytidylic (pIpC), with and without Dox, were first prepared, achieving an encapsulation efficiency >75% and a size <140 nm. In vitro studies identified that LNP pIpC was superior to CpG at activating bone marrow-derived immune cell populations (dendritic cells and macrophages) with minimal toxicity. It was also observed that the pIpC formulation can skew macrophage polarization toward the antitumorigenic M1 phenotype and increase macrophage phagocytosis of cancer cells. Upon intratumoral administration, pIpC Dox LNPs led to significant immune cell infiltration and activation. In survival models, the inclusion of Dox into pIpC LNP improved mice survival compared to control. However, addition of Dox did not show significant improvement in mice’s survival compared to singly formulated pIpC LNP. This study has illustrated the potential of pIpC LNP formulations in prospective GBM immunotherapeutic regimes. Future studies will focus on optimizing dosage regimen and/or combination with other modalities, including the standard of care (temozolomide), immune checkpoint blockade, or cancer vaccines.

## 1 Introduction

Despite extensive research, glioblastoma (GBM) remains one of the most devastating and prevalent types of brain cancer, constituting approximately half of all brain tumors.^1^ Even with the current standard of care, the prognosis remains poor, with a median overall survival of only 14.6 months and a 5-year survival rate of less than 10%.^2^

In the era of immunotherapy, immunogenic cell death (ICD)-inducing drugs are gaining momentum for their use in cancer treatment.^3^ Many conventional therapies have already been shown to induce ICD^4^ while others are currently being tested for their potential to do so.^5^ ICD occurs when certain types of chemotherapies, such as anthracyclines, are given at high dosages. Mechanistically, ICD is mediated through the release of damage-associated molecular patterns from dying cancer cells, causing activation of antigen-presenting cells (APC), such as macrophages and dendritic cells.^6^ Through this process, ICD-inducing drugs have been shown to modulate the tumor microenvironment toward an anti-tumor landscape.^7^ Among the most potent ICD-inducing drugs is doxorubicin (Dox), and preclinical studies have shown reduced tumor progression and prolonged survival in brain tumor-bearing rats treated with Dox.^8^ However, a phase II clinical trial using intravenously administered PEGylated liposomal Dox either alone or in combination with orally administered Tamoxifen showed only moderate success in clinical GBM.^9^

This low efficacy may be due to the profound immunosuppression associated with GBM, limiting the ICD effect of Dox.^10^ Immune adjuvants offer an elegant solution to this challenge. Among immune adjuvants, Toll-like receptor (TLR) agonists have shown great potential for cancer immunotherapy, but few have entered clinical trials.^11^ In general, most studies focus on their use in vaccines.^12−14^ However, this approach fails to acknowledge their endogenous anti-tumoral potential.^15^ Notably, the National Cancer Institute listed them among the 12 immunotherapeutic agents with potential to cure cancer.^16^ Particularly, the TLR3 agonist polyinosinic/polycytidylic acid (pIpC) has been shown to be effective against GBM in animal studies.^17^ Due to promising results, a phase I/II trial was set out to determine the effectiveness of pIpC in combination with radiotherapy. In this study it was demonstrated that pIpC was well tolerated and may be a suitable candidate for use in combined regimes.^18^ Another promising agent against GBM is the TLR9 agonist CpG, which entered a phase I clinical trial for patients with recurrent GBM.^19^

Combining drugs is an established means to enhance efficacy; however, it is likely that different modalities will have distinct kinetics and distribution dependent on molecular properties. This is especially relevant with combinations of drug and adjuvant, which are typically small and hydrophobic and large and hydrophilic, respectively. Through coformulation, it is possible to combine these disparate molecules and ensure simultaneous delivery to the same cell. Over the last decades, lipid-based nanoparticles (LNPs) have emerged as an effective delivery platform for both hydrophilic and hydrophobic molecules.^20^

A key advantage of lipid nanoparticles is their ability to increase cellular uptake of various anticancer agents by immune cells.^21,22^ Facilitating cellular uptake is particularly important with regards to endosomal active TLRs such as TLRs 3, 7/8, and 9 and cytosolically restricted RIG-I-like receptors (RLR) such as the retinoic acid-inducible gene I (RIG-I) and the melanoma differentiation-associated protein 5 (mda-5).^23^ Though lipid nanoparticles have shown promising results as delivery platforms for multiple types of tumors,^24,25^ there is little evidence of their application in the treatment of GBM. A significant hurdle in the development of therapies for GBM is the presence of the blood-brain barrier, which excludes many drug candidates.^26,27^ Direct intracranial injection into the tumor is the best means to achieve high local drug concentration; however, this technique has yet to be translated. While technically challenging, this approach is clinically feasible and can be performed via a biopsy needle or simultaneously with tumor resection. Such an approach may offer hope for otherwise poorly treated disease.

This study seeks to develop a safe single drug delivery system for the simultaneous local delivery of a TLR agonist as an immune adjuvant and the chemotherapeutic drug Dox for GBM therapy. We selected lipid nanoparticles as a delivery platform and will test the therapy in vitro on GBM and immune cell cultures and in vivo in an orthotopic GBM mouse model. The changes in tumor microenvironment will be characterized by flow cytometry, and LNPs will be tested therapeutically in tumor-bearing mice. We hypothesize that the combination of an ICD-inducing drug and an immune adjuvant in an LNP formulation will result in the establishment of an antitumorigenic state marked by increased immune cell infiltration. When administered in vivo, this formulation will result in tumor regression and prolonged survival.

## 2 Materials and Methods

### 2.1 Materials

Quant-it Ribogreen was purchased from Thermofisher. Amicon Ultra 30KDa MWCO filter units were purchased from Merck Millipore. siRNA duplexes with the siNEG sequence UGCGCUACGAUCGACGAUG were obtained from Eurogentec. Poly inosinic/polycytidylic acid (pIpC) was purchased from Bio Techne. The lipids used for LNP preparation were purchased as follows:1,2-Dioleoyl-*sn*-glycero-3-phosphoethanolamine (DOPE) from Lipoid, cholesterol from Sigma-Aldrich, Dlin-MC3-DMA from Bybit, and *N*-palmitoyl-sphingosine-1-succinyl [methoxy (polyethylene glycol) 2000] (C16 PEG 2000 Ceramide) from Avanti. Fisherbrand Sterile Cell strainer 70 μm mesh size and StemPro Accutase were purchased from FisherScientific. Antimouse fluorophore conjugated antibodies CD45 PB (30-F11), CD86 BV421 (PO3), MHCII – BV605 (M5), CD11c BV785 (N418), CD206 FITC (C068C2), CD40 PE (3/23), F4/80 PerCP (QA17A29), CD11b APC (M1/70), MHC I APC (SF1- 1.1), CD45 APC Cy7 (30-F11), Zombie Aqua fixable viability dye, TruStain FcX PLUS, Precision Count Beads, granulocyte- macrophage colony- stimulating factor (GM-CSF), macrophage colony-stimulating factor (M-CSF), Interleukin 4 (IL 4), and Interferon gamma (IFNγ) were obtained from BioLegend. Compensation for multicolor flow cytometry was performed utilizing UltraComp eBeads and ARC amine reactive beads (both Thermofisher). Tissue culture reagents: fetal calf serum|fetal calve serum (FCS), Trypsin EDTA, GlutaMAX, Advanced RPMI 1640 media, RPMI 1640 media, phosphate buffer saline (PBS), and penicillin–streptomycin were from Gibco, ThermoFisher Scientific. Unless stated otherwise, all chemical reagents were purchased from Sigma-Aldrich.

### 2.2 Methods

#### 2.2.1 Cancer Cell Line Culture

GL261 murine GBM cells expressing luciferase were cultured in advanced RPMI media containing 10% of total volume FCS, 1% GlutaMAX, and 1% penicillin/streptomycin antibiotics. All cells were maintained in an incubator at 37 °C in humidified air containing 5% CO_2_.

#### 2.2.2 Animals

All animal experiments were conducted in agreement with the existing personal and project licenses granted by the UK Home Office and in accordance with the UKCCCR Guidelines (1998). For in vivo experiments, mice were purchased from Charles River, UK. For bone marrow isolation, 3–6 week old male mice were used. For immune modulation and therapy studies, 4–6 week old male mice were used.

#### 2.2.3 LNPs Preparations

**Scheme 1:**
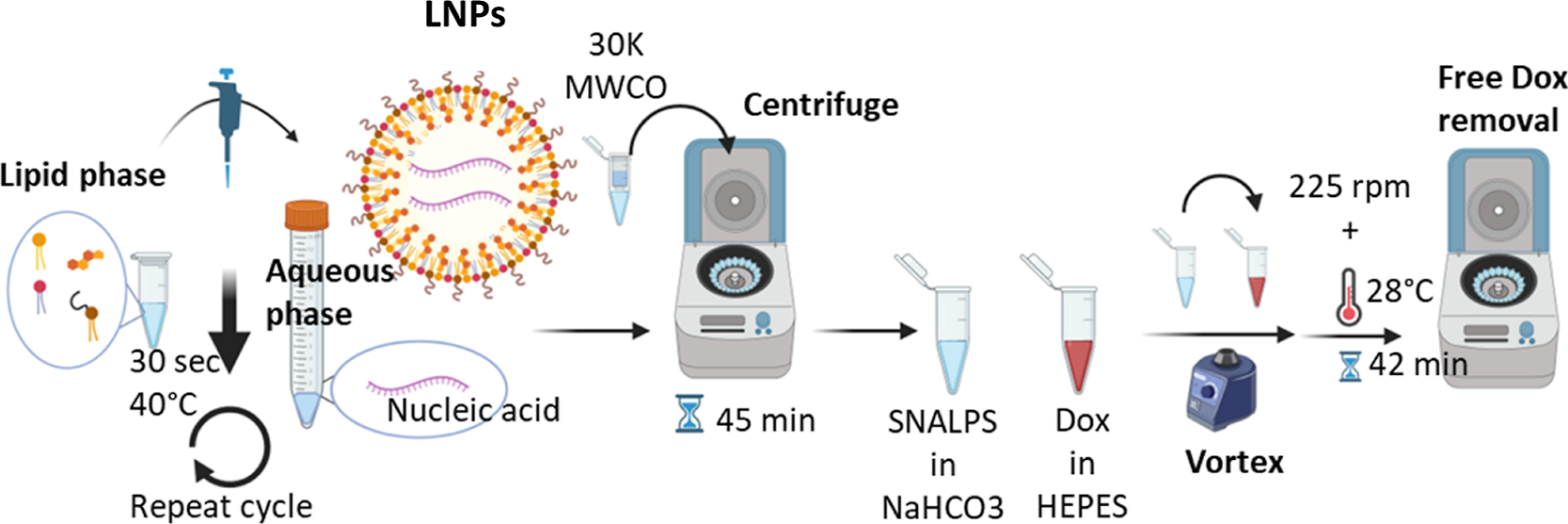
Preparation of LNPs Containing Nucleic Acid Immune Adjuvants With or Without Doxorubicin LNPs were prepared using the ethanol injection method. A lipid phase was created using 4 different lipids and citrate buffer, while the aqueous phase was made using citrate buffer and nucleic acid. After 5 min incubation at 60 °c (lipid phase) or 40 °C (aqueous phase), respectively, the lipid phase was added to the aqueous phase under strong vortex. The process was repeated until the aqueous phase was fully incorporated into the lipid phase. To allow spontaneous formulation, lnps were incubated before ethanol was removed via nitrogen flux to 100 μL. After buffer exchange, dox was added and incubated. for final buffer exchange, lnps were resuspended in hepes buffer after centrifugation.

LNPs were prepared using the ethanol dilution method, as described in Scheme 1. Dlin MC3 DMA, cholesterol, DOPE, and C16PEG2000 stock solutions were prepared by dissolving the lipids in absolute ethanol to a final concentration of 2 mg lipid/ml ethanol. C16 PEG2000 concentration was 4 mg/mL. To prepare the LNPs, a lipid solution and an aqueous solution were made in separate tubes. For the lipid solution, Dlin MC3 DMA, cholesterol, DOPE, and C16PEG 2000 stock solutions were mixed together before 10% of total lipid volume citrate buffer (20 mM, pH4) was added. The molar percentages of each component were 42.1%, 39.1%, 13.45%, and 5.28%, respectively. For the aqueous solution, 5 μg nucleic acid was added to 100 μL citrate buffer (20 mM, pH4). The ionizable lipid: nucleic acid ratio was 10:1 w/w. After prewarming the lipid solution to 60 °C and the aqueous solution at 40 °C for 5 min, 10 μL of the lipid solution was added to the aqueous one. The aqueous phase was then strongly vortexed for 10s before both solutions were again incubated at 60 and 40 °C, respectively, for 30s. The cycle was repeated until all the lipid solution was consumed. The new solution was then incubated at 40 °C for 1 h before the ethanol was removed under nitrogen flush to a final volume of 100 μL.

#### 2.2.4 Dox Loading of LNPs

For buffer exchange, LNPs incorporating nucleic acids were transferred to an Amicon Ultra 30 K MWCO filter unit and centrifuged for 45 min at 14,000 rpm and 4 °C. LNPs were resuspended in 200 μL of sodium bicarbonate buffer (0.5 M, pH8) and transferred to an Eppendorf tube. In a second Eppendorf tube, Dox (1:10 Dox: total lipid mass ratio) was added to 200 μL of HEPES buffer (pH7). Dox solution was transferred to LNPs suspension, vortexed, and incubated for 42 min at 28 °C and 225 rpm. Free DOX was removed via centrifugation in a 30 K MWCO Amicon tube at 14,000 rpm and 4 °C. LNPs were resuspended in citrate buffer or HEPES buffer (0.5 M, pH7) for in vitro and in vivo studies, respectively.

#### 2.2.5 Determination of Physicochemical Characteristics of LNPs

LNPs were characterized with the Zetasizer Nano ZS Series (Malvern Instruments, Southborough, MA) at 25 °C using dynamic light scattering. After preparation of SNALPs, 20 μL of sample was added to 1 mL of 0.15X PBS. The suspension was transferred to a disposable capillary Zeta cell. The results are averages of 10 runs, each of which was performed in triplicate. Mean particle size is represented by Zeta Average (d.nm), and size distribution is represented by Polydispersity Index (PDI).

#### 2.2.6 Determination of RNA/DNA Encapsulation Efficiency

The encapsulation efficiency of the nucleic acid was quantified indirectly using the Quant-iT RiboGreen assay following the manufacturer’s protocol. For LNP sample analysis, 2 μL LNPs were suspended in either 48 μL PBS or 48 μL 5% Triton X- 100/PBS solution and mixed by gently pipetting up and down. Simultaneously, standard curves were constructed ranging from 1.25 to 20 ng/μL soluble nucleic acid in PBS or 5% Triton X- 100/PBS solution. LNPs and standards in PBS were then incubated for 45 min at room temperature, while standards and LNPs in Triton X-100 were incubated at 37 °C. After incubation time, RiboGreen reagent was added to each well as per manufacturer instructions. Both the standard curves and samples were analyzed by measuring the fluorescence with a plate reader at excitation and emission 480 m and 520 nm, respectively (BMG LABTECH, FLUOstar Omega). The coefficient of determination (*R*_2_) of the standard curves was ≥0.95 for all nucleic acids.

The encapsulation efficiency was then calculated using the formula
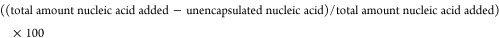


#### 2.2.7 EE % of Dox

To determine the EE % of Dox. EE % was calculated indirectly by measuring the amount of unencapsulated Dox in the supernatant. A calibration curve, ranging from 2.61 to 20.88 μg/μL, was constructed by making a serial dilution of Dox in Tris buffered saline in a 96-well plate. The absorbance was measured via UV–vis spectrofluorometer plate reader (BMG LABTECH, FLUOstar Omega) at excitation and emission of 300 and 590 nm, respectively.

The encapsulation efficiency was then calculated by using the following formula
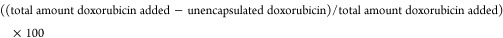


#### 2.2.8 MTT Assay

The MTT assay was performed to assess cell metabolic activity as a measure of their viability. On day 0, 5k GL261.luc cells in 100 μL of media were seeded in a 96 well plate and incubated at 37 °C with 5% CO_2_. After 24 h, media was replaced with media containing nucleic acid or Dox at a range of concentrations: 0.01–100 μg/mL (for nucleic acids) or 0.01–100 μM (for Dox), and cells were incubated for 48 h. Media was removed and replaced with 120 μL of MTT at a final MTT concentration of 0.83 mg/mL. Following 4 h incubation, MTT solution was exchanged for 200 μL of DMSO, and absorbance was measured at 570 nm using a UV–vis spectrofluorometer (BMG LABTECH, FLUOstar Omega).

Cell viability was expressed using the following formula
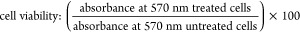


#### 2.2.9 Dendritic Cell and Macrophage Culture from Bone Marrow

On day 0, cells were isolated from the femur and tibiae of 3–6 w old mice. After removing all tissue from the bones, both epiphyses were cut off with a scalpel to expose the marrow. The bone marrow was then flushed with RPMI 1640 media, containing 10% FCS, 1% Glutamax, and 1% penicillin/streptomycin, referred to as complete RPMI media, until the bones appeared translucent. The flow through was filtered using a cell strainer before being centrifuged at 700rcf at 4 °C for 5 min to collect cells. The supernatant was discarded, and 500 μL of 1x red cell lysis buffer was added to the cell pellet. The solution was then resuspended in 5 mL of complete RPMI to stop further lysis before centrifugation as above. Cell pellets were resuspended in complete RPMI media containing 10 ng/mL M- CSF (for macrophage differentiation) or GM-CSF (for DC differentiation). Cells were seeded into 24 well plates at a seeding density of 500000 cells/well in 500 μL of complete RPMI media. On day 3, media was supplemented with the following cytokines depending on the cell phenotype to be obtained: (i) no additional cytokines to obtain M0 macrophages, (ii) 50 ng/mL IL 4 to obtain M2 macrophages, or (iii) 50 ng/mL IFN-γ to obtain DCs and M1 macrophages. On day 4, different treatments were added to cells at a concentration of 1 μg/μL and incubated for further 48 h before analysis.

#### 2.2.10 Assessment of Differentiation Markers on Immune Cells by Flow Cytometry

Cells were analyzed for changes in surface marker expression in response to immune adjuvant treatment using flow cytometry. Cells were detached from the plates by the addition of 500 μL of ice-cold cell dissociation buffer to each well and repeated pipetting before being transferred to Eppendorf tubes. After centrifugation at 1750 rpm for 5 min at 4 °C, the supernatant was discarded, and cells were washed once with 100 μL of PBS. Pellets were resuspended in 100 μL PBS and transferred to a 96 well plate. To assess viability, cells were stained with 50 μL/well Zombie Aqua Fixable Viability Kit (diluted 1:250 with 1xPBS) for 20 min at room temperature in the dark. After washing as above, cells were incubated with 50 μL of TruStain FcX PLUS (1:200 antibody/PBS dilution ratio) for 30 min on ice. Following one washing step, samples on the 96 well plate were incubated with antibodies of interest at 1:200 dilution in 50 μL for 30 min on ice. After two additional washing steps, cells were resuspended in 200 μL of PBS and transferred to FACS tubes before 10 k viable cells were acquired with a BD FACSCelesta Cell Analyzer. The data was analyzed using the FlowJo Software (TreeStar, Inc.). The plots were analyzed after excluding debris using forward–sideward scatter gating, then selecting singlets through forward scatter gating and a selection of viable cells based on Zombie Aqua intensity. CD86 and CD40 cell surface expression was analyzed using the Mean Fluorescent Intensity (MFI) of each marker, while the M2 → M1 switch was assessed through gating for percentage positive cells (M1: F4/80^+^, CD11b^+^, MHCII^+^, CD206^–^ M2: F4/80^+^, CD11b^+^, MHCII^+^, and CD206^+^).

#### 2.2.11 Assessment of MHC Class I Upregulation on Gl261 Cells In Vitro by Flow Cytometry

GL261 luc cells were seeded on day 0 on a 24 well plate at a seeding density of 75,000 cells per 500 μL of media. After 24 h of incubation, media was discarded, cells were washed with PBS, and different formulations containing the TLR9 agonist pIpC soluble in LNP, with and without addition of Dox, were added to the cells at a concentration of 7.5 μg/mL of nucleic acid and 0.18 μM, respectively. As a control, siNEG LNP with and without Dox was used at equivalent nucleic acid and Dox concentrations as controls. Following 48 h incubation, cells were trypsinized and analyzed via flow cytometry, as described for the immune cell analysis. Following one washing step, cells were stained with MHC class I (1:200 dilution) and incubated for 30 min at 4 °C in the dark. After incubation and washing, cells were transferred to FACS tubes and 20,000 viable cells acquired by a BD FACSCelesta Cell Analyzer. FlowJo Software (TreeStar, Inc.) was used for the analysis. After excluding debris by FSC vs SSC gating, single cells were chosen based on FSC-A vs FSC-H gating. Viable cells were chosen based on the intensity of ZombieAqua. MHC class I expression was assessed through the mean fluorescence intensity on viable cells only.

#### 2.2.12 GL261 glioblastoma/J774 Macrophage Coculture

On day 0, 70 k cells GL261 or J774 were seeded on a 24 well plate in 500 μL media. In the case of GL261, cells were stained with 5 μmol of DiD dye prior to seeding. 24 h later, media was discarded, cells were washed, and different formulations were added at nucleic acid and Dox concentrations of 7.5 μg/mL and/or 0.18 μM Dox, respectively. Following 48 h incubation, J774 macrophages were added to GL261 cells at a 1:1 number ratio. After 6 h of coculture, cells were detached using StemProAccutase and gentle scraping. For flow cytometric analysis, cells were centrifuged at 1750 rpm for 5 min, washed once with PBS, and transferred to a 96 well plate in 100 μL of PBS. For viability assessment, 50 μL of Zombie Aqua, diluted 1:250 with PBS, was added to each well and incubated in the dark at room temperature. Following one washing step, cells were stained with 50 μL of F4/80 antibody, diluted 1:200 with PBS. After 30 min incubation at 4 °C in the dark and washing once with PBS, cells were transferred to the FACS tube, and 20 k viable cells were acquired via a BD FACSCelesta Cell Analyzer. FlowJo Software (TreeStar, Inc.) was used for analysis. After excluding debris by FSC vs SSC gating, single cells were selected based on FSC-A vs FSC-H gating. Viable cells were determined based on the intensity of ZombieAqua. After selection of J774 macrophages, based on high F4/80 expression, MFI was used to determine engulfment of GL261 cancer cells.

#### 2.2.13 Preparation of the Orthotopic GL261.luc Mouse Model

Mice were injected stereotactically with 200,000 GL261.luc cells suspended in 2 μL of PBS using a Hamilton Syringe (Harvard Apparatus, UK) with a 26-gauge needle. The consistency was ensured by following a precise intracranial injection technique. Animals were first anesthetized through isoflurane inhalation before the skull was opened and the bregma was identified. Based on this anatomical landmark, the stereotactic coordinates for injection were 0.5 mm anterior, 1.5 mm lateral, and 2.5 mm deep. After opening the skull with a drill, the cell suspension was injected at a rate of 0.2 μL of cell suspension/min. The injection volume and speed were kept constant for all mice to avoid variability in the dispersion of the formulation within brain tissue. Additionally, our LNP formulations were prepared consistently before each injection, following a standard protocol. Size, PDI, zeta potential, and %EE were checked to ensure uniform dosing. To monitor tumor growth, mice were imaged on days 3, 6, 9, 12, and 14 after tumor inoculation using quantitative in vivo bioluminescence imaging (IVIS Lumina III, PerkinElmer, UK). During anesthesia with isoflurane inhalation, mice were injected subcutaneously with 150 mg/kg D-luciferin. The images were recorded 10 min after injection and analyzed using living Image software (PerkinElmers, UK). The bioluminescence signal is expressed as total flux (photon/s).

#### 2.2.14 In Vivo Studies

After confirmation of tumor growth via in vivo bioluminescence imaging, mice were injected intracranially with LNP in a total volume of 10 μL of HEPES buffer (0.5 M, pH7). Following anesthesia via isoflurane inhalation, the skull was reopened and the injection site identified. The same burr hole was used for the treatment injection at an injection rate of 0.2 μL/min.

#### 2.2.15 Immune Infiltration and Activation Studies

Mice (*n* = 3 per group) received 5 μL of one i.t. injection of HEPES buffer (sham), siNEG LNP, siNEG Dox LNP, CpG LNP, pIpC LNP, or pIpC Dox LNP at nucleic acid or Dox doses of 375 μg/kg and 1180.5 μg/kg, respectively. Three days later, the mice were sacrificed. After intraperitoneal lethal injection of pentobarbital sodium and loss of consciousness, mice were subjected to transcardiac perfusion with a total of 25 mL of 0.5% saline to remove red blood cells from the brain, which may hinder later flow cytometry analysis. Tumors were then extracted and incubated for 1 h at 37° in 6 μL/mg digestion buffer. After 2x washing in PBS, 100 μL of cell suspension in PBS was transferred to a 96 well plate. Zombie Aqua was used as a viability dye at a dilution ratio of 1:250 with PBS. 50 μL of the diluted dye was added to each well and incubated in the dark at room temperature for 30 min. Following one washing step, cells were stained with 50 μL of antibody mix, each of which antibody was diluted 1:200 with PBS. After 30 min of incubation at 4 °C in the dark and washing once with PBS, 175 μL of cell suspension from each well was transferred to the FACS tube. After addition of 25 μL Precision Count Beads to each tube, 500,000 viable cells were acquired via BD FACSCelesta Cell Analyzer. FlowJo Software (TreeStar, Inc.) was used for analysis. After excluding debris by FSC vs SSC gating, single cells were selected based on FSC-A vs FSC-H gating. Viable cells were determined based on the intensity of ZombieAqua. Immune cells were identified as CD45+ cells. From those, tumor-associated macrophages were selected as the CD45^high^/CD11b ^high^ population, dendritic cells as CD45^high^/11c ^high^ and T Cells as CD45^high^/CD3 ^high^. After selection of T cells, this population was further gated for CD4+ T helper cells as CD45^high^/CD3 ^high^/CD4 ^high^/CD8^low^, while CD8+ cytotoxic T cells were chosen based on CD45^high^/CD3 ^high^/CD8 ^high^/CD4^low^ expression levels. Activation marker expression was analyzed through median fluorescence intensity on all immune cell subpopulations.

#### 2.2.16 Survival Studies

5–6 weeks old C57BL/6 mice (5–6 weeks old) were implanted with GL261, as described above. Seven and 14 days after tumor implantation, mice (*n* = 8 per group) received 5 μL of i.t. injection of HEPES buffer (sham), soluble pIpC, pIpC LNP, siNEG/Dox LNP, or pIpC/Dox LNP at nucleic acid or Dox doses of 0.45 and 1.53 mg/kg, respectively. Mice were monitored for tumor growth via BLI imaging and changes in body weight twice a week. Mice were sacrificed when body weight loss exceeded 20%, experienced any signs of stress for over 24 h, or experienced 15% weight loss and any stress signs combined. Kaplan–Meier survival curves were obtained for mice with and without treatments.

#### 2.2.17 Statistical Analysis

GraphPad Prism 9 was used for the numerical analysis. Statistical analysis was carried out utilizing one-way ANOVA followed by a Tukey test. For survival analysis, the Mantel–Cox test was used. The statistical test used is indicated in each figure caption. For each group, apart from the macrophage polarization and survival studies, *n* = 3 samples or mice were used. For macrophage polarization, *n* = 4 mice per group were used. For survival studies, *n* = 8 mice per group were utilized. Flow cytometry data was analyzed using FlowJo Software (Version 10.8, TreeStar).

## 3 Results

### 3.1 In Vitro Studies

#### 3.1.1 RNA/DNA Dox Combination Can be Successfully Encapsulated in Lipid Nanoparticles and Shows Favorable Physicochemical Characteristics

LNPs were prepared according to a previously reported method for encapsulation of siRNA together with mRNA.^28^ For encapsulation of Dox, a pH gradient method was employed.^29^ As shown in Table 1, all formulations were <150 d.nm, the largest being pIpC Dox LNPs (Figure S1). The smallest particle was CpG LNP. In general, an increase in size was observed after encapsulating Dox in particles which already contained a nucleic acid (CpG LNP < pIpC LNP < CpG Dox LNP < pIpC LNP). The physicochemical analysis further showed that all particles were monodisperse, indicated by PDI <0.2.

**Table 1:** Physicochemical Characterization of Different LNP Formulations^e^

LNP formulation	size (d.nm)^a^^,^^d^	PDI^a^^,^^d^	zeta Potential (mV)^a^^,^^d^	%EE of nucleic acid^b^^,^^d^	%EE of doxorubicin^c^^,^^d^
CpG	115.63 ± 0.68	0.11 ± 0.01	–1.21 ± 1.03	98.67 ± 1.40	
CpG/Dox	140.94 ± 1.33	0.15 ± 0.00	–6.47 ± 0.20		80.14 ± 3.73
plpC	120.5 ± 0.44	0.08 ± 0.03	–7.18 ± 4.86	78.16 ± 3.80	
plpC/Dox	144.63 ± 0.67	0.17 ± 0.01	–9.80 ± 0.54		75.45 ± 2.87

a Measured by dynamic light scattering.

b Encapsulation efficiency (% EE) calculated as a percentage of total nucleic acid initially added determined by the ribogreen assay.

c % EE calculated as percentage of total doxorubicin initially added determined by spectrofluorimetry.

d Expressed as mean ± SD (*n* = 3).

e % EE calculated as percentage of total doxorubicin initially added (51 μg) determined by direct quantification of Dox using fluorimetry.

To confirm the successful encapsulation of nucleic acid and Dox, the encapsulation efficiency (EE %) was calculated using standard curves presented in Figure S2. Table 1 shows the encapsulation efficiencies for the different formulations. CpG, a molecule that is smaller in size compared to pIpC, showed the highest encapsulation efficiency of ∼100%. pIpC encapsulation efficiency reached approximately 80%. Encapsulation efficiency for Dox was consistently above 75%. For CpG Dox LNP, the encapsulation efficiency was ∼80%, while pIpC Dox LNP resulted in ∼75% encapsulation. Dox was demonstrated to be stably incorporated into LNP with minimal leakage after 6 h and <20% release after 24 h dialysis at 37 °C with stirring (Figure S3).

#### 3.1.2 Nucleic Acid Immune Adjuvants Show Dose-Dependent Cytotoxicity in the Cancer Cell Line and Bone Marrow-Derived Immune Cells

In the next step, cell viability upon the addition of different formulations to GL261 and immune cells was assessed. For all cell types, a serial dilution between 0.001 and 10 μg/mL of nucleic acid was prepared. The range was chosen based on the dosages that would be used in subsequent in vitro and in vivo studies. In general, neither a significant difference between cancer and immune cell cytotoxicity nor significant differences between treatments were observed (Figures S4–S6). Even at dosages as high as 10 μg/mL, viability remained at approximately 70% in all cases. GL261 brain tumor cells showed the lowest cell viability of all cell lines, where the addition of 10 μg/mL pIpC LNP decreased to ∼70%. The lowest reduction in cell viability was observed in dendritic cells after addition of 10 μg/mL siNEG LNP, where viability remained at ∼85%. In contrast to GL261 brain tumor cells, dendritic cells were most sensitive to CpG LNP, showing a decrease to ∼78% cell viability. Similar to GL261 brain tumor cells, M0 macrophages were sensitive to treatment with pIpC (∼76%), while at dosages of 10 μg/mL the lowest toxicity was observed in treatment with soluble CpG (∼82%).

#### 3.1.3 pIpC LNP Results in Potent Activation of Immune Cells In Vitro

To assess the effect of LNP formulations on macrophages and dendritic cells, bone marrow cells were cultured in the relevant cytokine cocktail, as described in the methods. After 3 days of incubation with the treatments, cells were analyzed for successful differentiation into the desired cell type via flow cytometry (Figure S7). Dendritic cells were identified through high expression of CD11c, M0 as low expression of MHCII and CD206, M1 as MHCII high and CD206 low, and M2 as MHCII and CD206 high. The highest obtained percentage of the desired cell type was M1 macrophages, where on average 92% of the cells showed a high expression of MHCII and low expression of CD206. Dendritic cells showed a similarly high percentage of differentiation, as on average, 82% of the cells were DCs. In contrast, the least efficient differentiation protocol was used for M2 macrophages. Here, only 28% of the viable population acquired the M2 macrophage phenotype. Where no cytokines were added to obtain M0 macrophages, ∼52% of the viable population showed the expression of markers characteristic of M0 macrophages.

**Figure 1:**
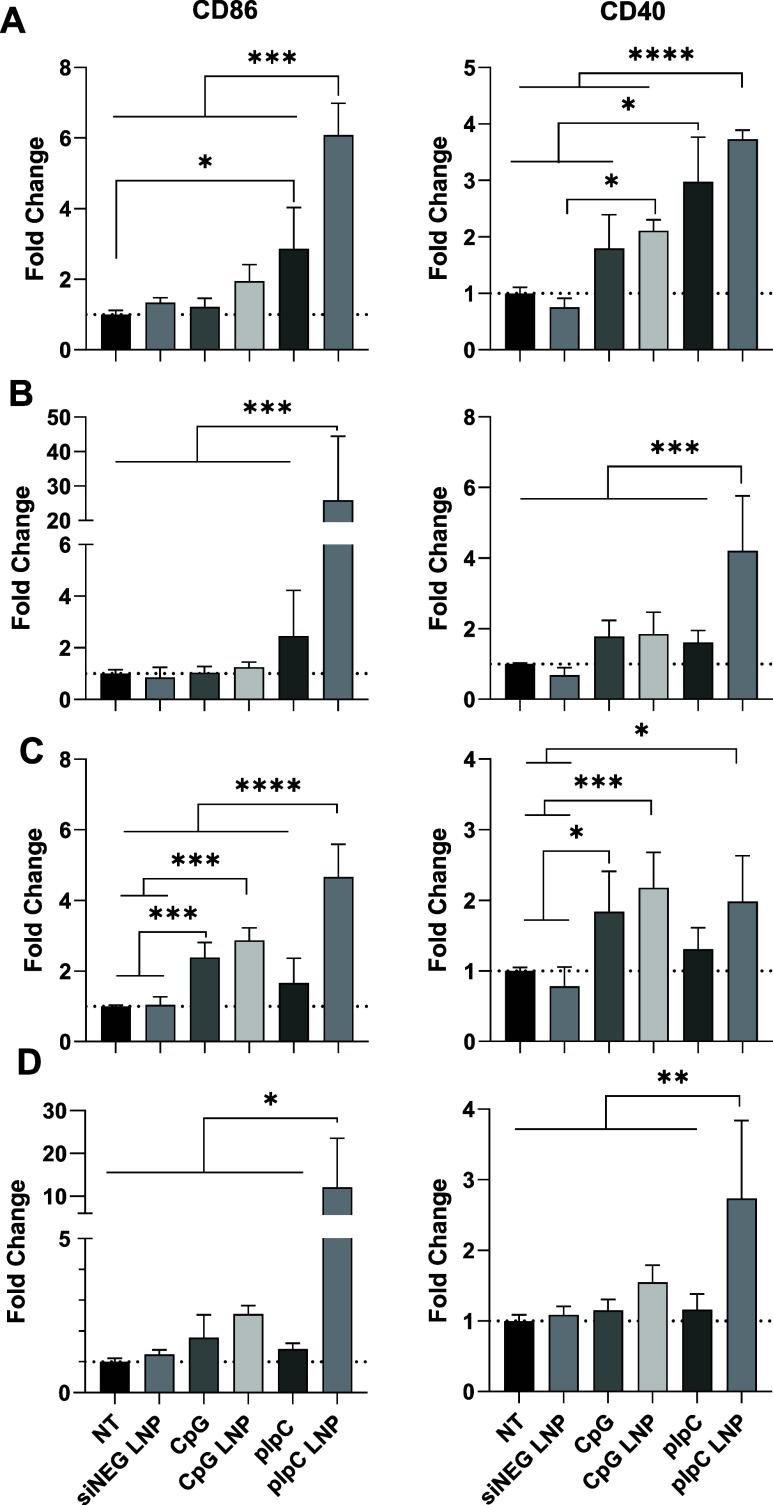
plpC and CpG LNPs activate immune cells in vitro. Bone marrow cells were differentiated into (A) DCs, (B) M0, (C) M1, or (D) M2 macrophages. On day 3 post differentiation, cells were treated with 10 μg/mL of either an unformulated TLR agonist (CpG, pIpC) or an LNP-formulated agonist (CpG LNP, pIpC LNP). A group was left untreated (NT) or treated with a LNP containing siNEG (siNEG LNP) as a control for nonspecific activation. Activation markers were assessed by flow cytometry and analyzed using FlowJo v10.8 Software. The fold change when compared to NT is plotted in each case. Data points represent mean and SD (*n* = 3 per group). Statistical analysis was carried out using one-way ANOVA followed by the Tukey test with significance of ****p < 0.0001, ***p < 0.0002, **p < 0.005, and *p < 0.05.

The immune cell populations were incubated with 10 μg/mL of either pIpC or CpG in soluble or LNP format for 48 h prior to analysis via flow cytometry. siNEG LNP was used as a negative control at the same concentration of nucleic acid to confirm that activation does not result from the LNP but from the nucleic acid immune adjuvants. pIpC LNP achieved the highest increase in MFI of CD86 and CD40 activation marker expression for all cell types (Figure 1A–D). In DC, both pIpC soluble and pIpC LNP increased the MFI, with 6-fold and 4-fold increases observed for CD86 (pIpC LNP > pIpC soluble) and CD40 (pIpC soluble ≈ pIpC LNP), respectively (Figure 1A). M0 macrophages, which are nondistinguished, showed the highest upregulation of all bone marrow-derived immune cells after addition of pIpC LNP, resulting in ∼25- and ∼4-fold increases in CD86 and CD40 expression compared to control (Figure 1B). Other treatments did not significantly increase CD86/CD40 expression. The antitumorigenic M1 macrophages, unlike other immune cells, showed a marked increase in CD40 and CD86 upon treatments with CpG LNP as well as pIpC LNP (Figure 1C). Interestingly, both CpG soluble and CpG encapsulated in LNP were able to increase activation marker expression with no significant difference found between them. Like M0 macrophages, only pIpC LNP treatment resulted in CD40 and CD86 expression change in protumorigenic M2 macrophages, increasing expression by 12- and 2.7-fold, respectively (Figure 1D).

#### 3.1.4 Nucleic Acid Immune Adjuvants Favor M1 Macrophage Polarization

**Figure 2:**
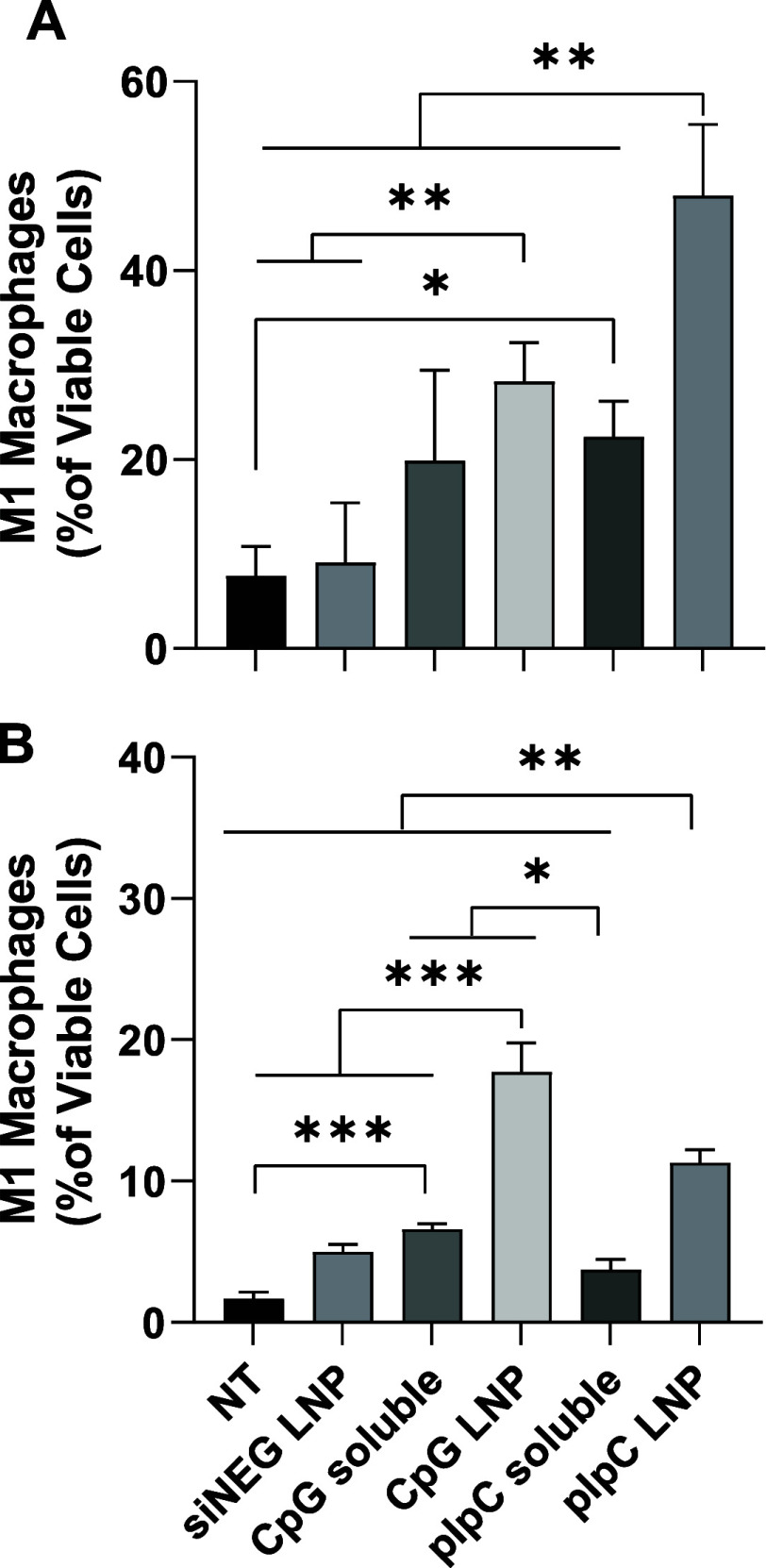
LNPs can polarize bone marrow-derived macrophages toward an immunostimulatory M1 phenotype. Cells isolated from mouse bone marrow were differentiated toward either an (A) M0 or (B) M2 phenotype by culturing in the presence of M-CSF or M-CSF plus IL-4, respectively. Following differentiation, cells were treated with immune adjuvants (CpG or pIpC) either in the soluble form or formulated in LNP (soluble or LNP) at 1 μg/mL for 48 h. LNPs formulated with siNEG (siNEG LNP) were used as a negative control. Cells were harvested and stained for M1 macrophage markers before being acquired on a FACSCelesta. Viable cells were gated, and the percentage of M1 macrophages (MHCII+/CD206-) analyzed. Data points represent mean and SD (*n* = 4 per group). Statistical analysis was carried out using one-way ANOVA followed by a Tukey test with significance of ****p < 0.0001, ***p < 0.0002, **p < 0.0021, and *p < 0.0332.

In addition to activation of macrophages and DC, the TLR3 and TLR9 agonists have been reported to induce differentiation from M0 to M1 macrophages.^30,31^ Similar to the activation results, the highest differentiation from M0 to M1 macrophages was observed after treatment with 10 μg/mL pIpC LNP (Figures 2A and S8).

In this group, addition of pIpC LNP resulted in nearly a 7-fold increase in differentiation to M1 macrophages compared to no treatment. pIpC soluble, CpG soluble, and CpG LNP also resulted in higher percentages of M1 macrophages compared to no treatment, but the effect was approximately half that of pIpC LNP treatment. Interestingly, it was also observed that macrophages that had been differentiated into M2 macrophages could be repolarized to M1 macrophages, with CpG LNP having the strongest effect, followed by pIpC LNP, and then CpG soluble (Figure 2B). After CpG LNP treatment, M1 macrophages constituted over 15% of all macrophages, while culture in media resulted in only 1% M1 macrophages.

#### 3.1.5 pIpC in an LNP Formulation Leads to Highest Increase in MHC I Expression

**Figure 3:**
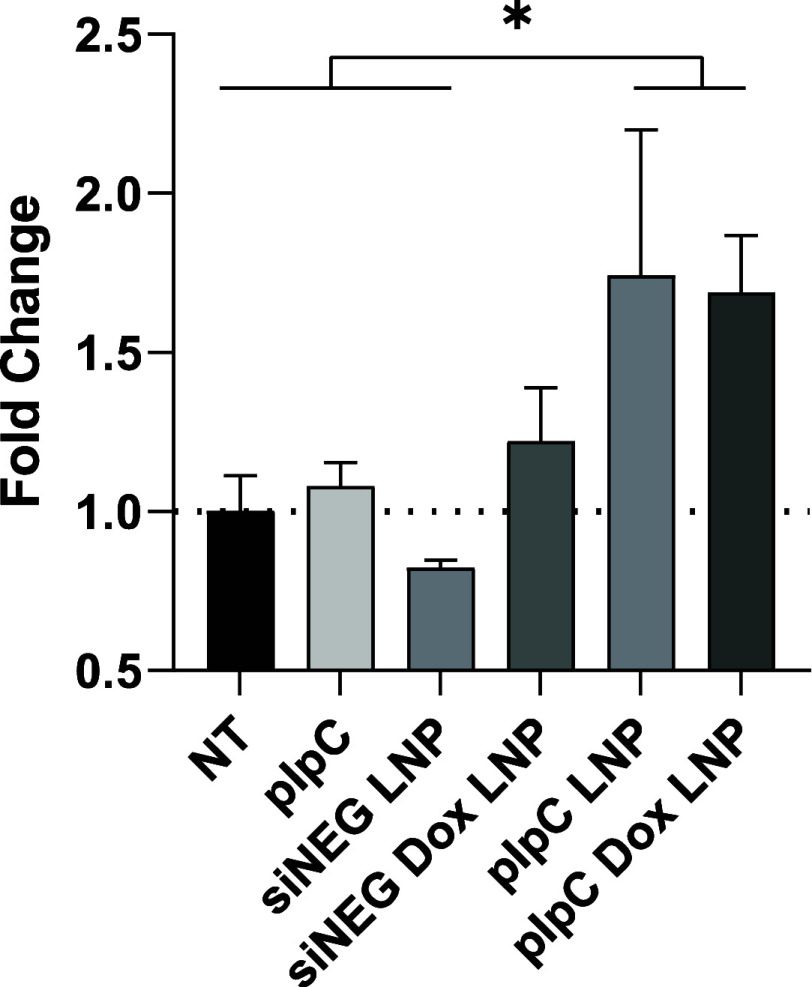
pIpC LNP and pIpC DOX increase MHC class I expression on GL261 cells in vitro. GL261 cells were treated with either soluble pIpC (pIpC), LNP-formulated pIpC (LNP pIpC), or LNP-formulated siNEG (siNEG LNP) at 1 μg/mL for 48 h. In some conditions, Dox was included at 1.1805 μg/g. An untreated group (NT) served as a negative control. Data was analyzed by flow cytometry using FlowJo Star v 10.8 software. Viable cells were first gated before MHC class I MFI was assessed. The fold change compared to NT is plotted. Data points represent mean and SD (*n* = 3 per group). Statistical analysis was carried out using one-way ANOVA followed by the Tukey test with significance of *p < 0.05.

MHC class I plays a pivotal role in antigen presentation but is downregulated in various types of cancer, such as GBM.^32^ A recent study found that MHC class I could be upregulated through liposomal Dox in a melanoma mouse model^33^ as well as through pIpC in a Gl261 cell culture.^34^ This study set out to determine whether Dox would also lead to upregulation on GBM cells and whether the effect could be further heightened by the addition of pIpC. GL261 cells were treated for 48 h before MHC class I expression assessment by flow cytometry. To allow incubation with Dox without causing complete cell death, 0.18 μM Dox was chosen as a dosage, as this molarity represents the IC_50_ value, determined by the previously performed MTT assay. The amount of nucleic acid was 10 μg/mL. Only pIpC LNP and Dox pIpC LNP significantly increased the MHC class I MFI with no difference observed between the two formulations (Figure 3). Considering that siNEG Dox LNP did not show increased MHC class I expression, these findings indicate that the observed upregulation was due to the presence of pIpC.

#### 3.1.6 pIpC LNP with and without Doxorubicin Increase GL261 Engulfment by Macrophages

**Figure 4:**
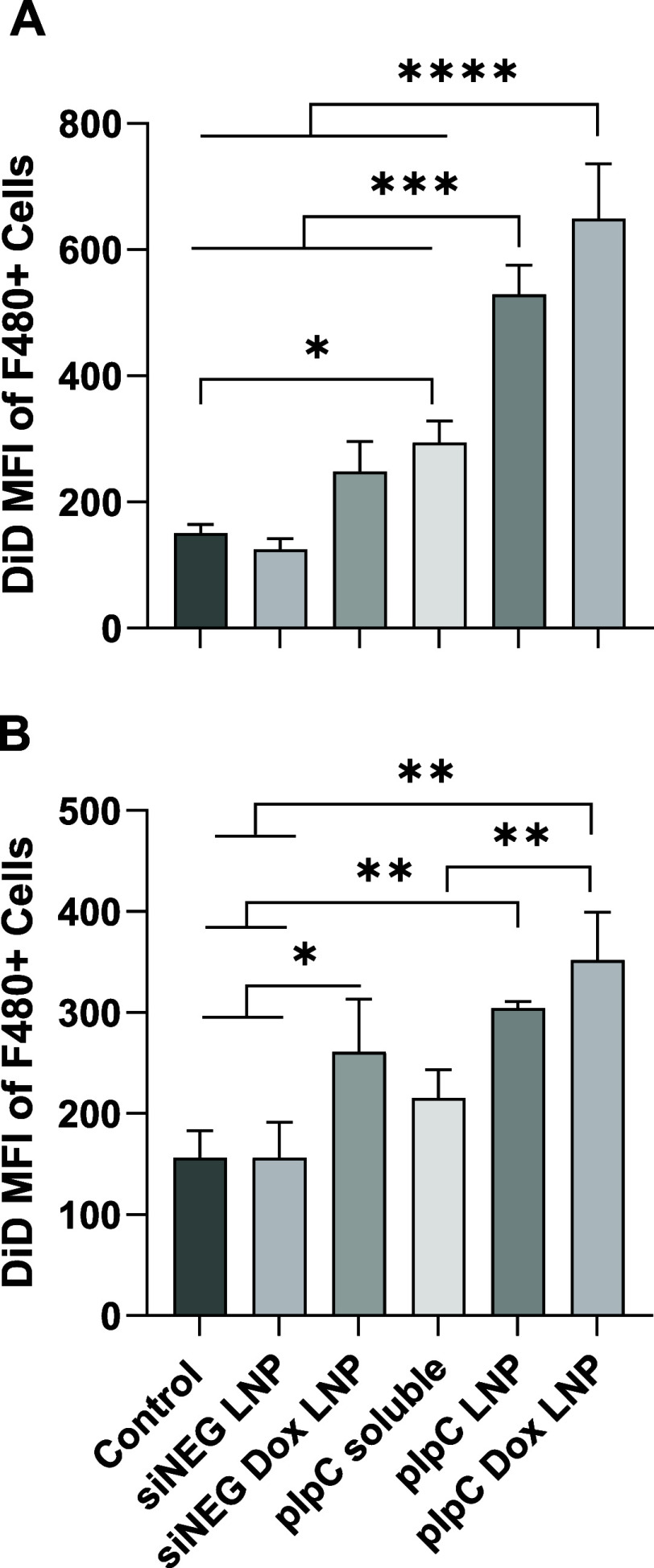
Treatment of J774 macrophages or GL261 with either pIpC, Dox, or combination LNP increases phagocytosis in vitro. To identify uptake, GL261 cells were first labeled with DiD (5uM) 24 h prior to use. Either (A) J774 macrophages or (B) labeled GL261 were treated with 1 μg/mL of soluble pIpC or pIpC formulated as an LNPs alone or in combination with 0.18 μM Dox. As a negative control, siNEG was formulated as an LNP (siNEG LNP). After 48 h of incubation, cells were harvested, and the treated cells were cocultured with the untreated cells at a 1:1 number ratio for 6 h. To identify macrophages in the coculture, cells were stained with the anti F480 monoclonal antibody before being acquired on a FACSCelesta flow cytometer. Viable cells were first gated before the J774 macrophage population was identified based on F480 expression. The mean fluorescence intensity of the DiD signal within the J774 population up taking GL261 cells (DiD+, F480+) is plotted above. Data points represent mean and SD (*n* = 3 per group). Statistical analysis was carried out using one-way ANOVA followed by the Tukey test with a significance of ****p < 0.0001, ***p < 0.0002, **p < 0.0021, and *p < 0.0332.

The dynamic between cancer cells and macrophages was assessed in a DiD-labeled GL261 and J774 macrophage coculture. Either of the cell types were treated with one of the formulations, Dox LNP, pIpC LNP, or pIpC Dox LNP, or the appropriate controls, for 24 h before cells were mixed at a 1:1 ratio, mimicking the GBM tumor microenvironment, for another 6 h. Overall, pretreatment of J774 cells as opposed to GL261 led to more efficient uptake of cancer cells by macrophages, as indicated by higher MFI values in Figure 4. Comparing the different treatments, pIpC Dox LNP followed by pIpC LNP resulted in the highest cancer cell phagocytosis by J774 cells. pIpC soluble and siNEG Dox LNP also resulted in GL261 cell phagocytosis, yet less efficiently compared to pIpC Dox LNP and pIpC LNP groups.

#### 3.1.7 pIpC Dox LNP Results in Highest Immune Cell Activation in the Tumor Microenvironment In Vivo and Improved Mice Survival

After promising in vitro results, the different formulations were intracranially injected into GL261 brain tumors on day 9 post tumor inoculation, mice were sacrificed 3 days later, and changes in the tumor microenvironment were assessed by flow cytometry (Figure S9).

**Figure 5:**
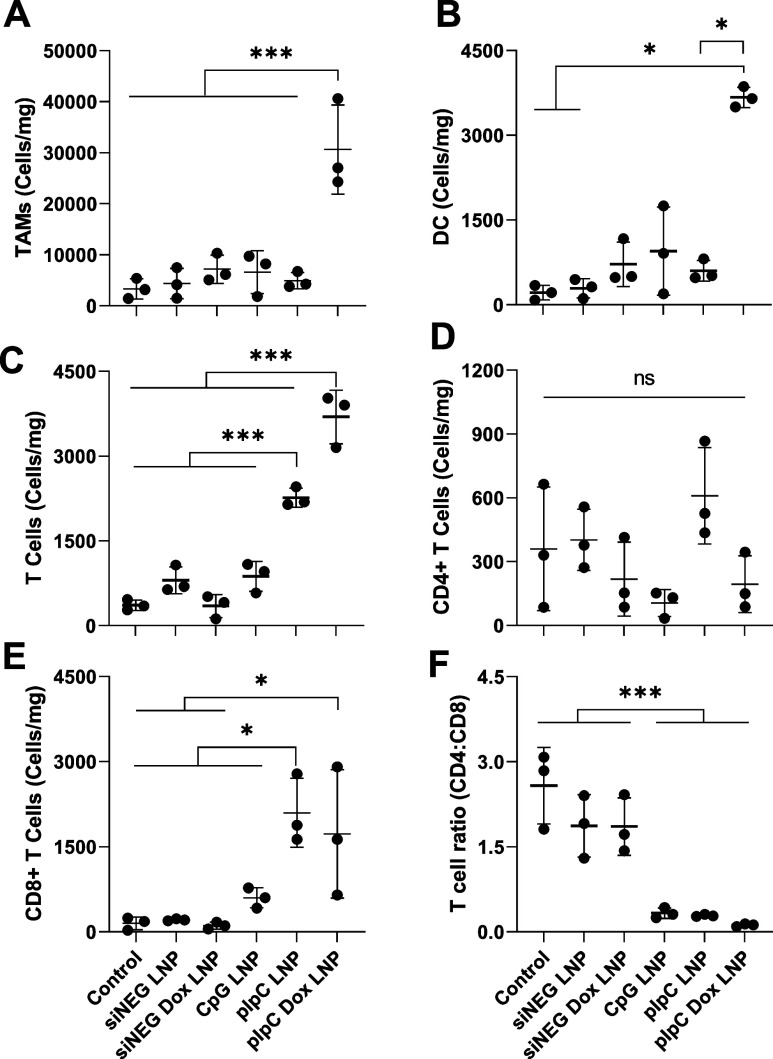
Combinatory pIpC Dox LNP therapy increases immune cell infiltration of GBM in vivo. Mice (C57BL/6, *n* = 3 per group) were implanted i.c. with GL261 on day 0, tumors were allowed to form, and at day 10 post implantation, the cranial burr hole was reopened, and mice were injected i.c. with LNP containing immunoadjuvant (CpG or pIpC) alone or coformulated with Dox at a dosage of 375 and 1180.5 μg/kg, respectively. LNPs formulated with siNEG (siNEG LNP) served as a negative control to account for the immunostimulatory properties of the formulation itself. Three days following administration of substances, mice were perfused with 0.9% saline, brains dissected, and single-cell suspension was obtained through physical maceration and enzymatic digestion. The phenotype of extracted cells was analyzed by flow cytometry, and extracted cells were identified as the following: (A) tumor-associated macrophages (TAMs, CD45^high^, CD11b^high^), (B) dendritic cells (DC) (CD45^high^, CD11c^high^), (C) total T cell counts (CD45^high^, CD3^high^), (D) CD4+ helper T cells (CD45^high^, CD3^high^,CD4^high^), and (E) CD8+ cytotoxic T cells (CD45^high^, CD3^high^,CD8^high^). Absolute cell counts were obtained by inclusion of a fixed volume of precision count beads into each stain; the value obtained was normalized to the tumor weight. T cell ratio was obtained by dividing the number of CD4 T cells by the number of CD8 T cells (F). Each data point represents a single animal mean and SD (*n* = 3 per group). Statistical analysis was carried out using one-way ANOVA followed by the Tukey test with significance of ****P < 0.0001, ***P0.0002, **P0.0021, and *P0.0332.

As shown in Figure 5, the pIpC LNP resulted in an increase in CD8 and total T cell numbers but did not affect any other immune cells. In contrast to these findings, the addition of Dox to pIpC LNP achieved an increase in all immune cells that were assessed; this is apparent when cell subsets as a percentage of leukocytes are analyzed (Figure S10).

Interestingly, CD4 T cells did not show any changes upon treatment (Figure 5D). CpG LNP only affected the CD4/CD8 T cell ratio, which was lowered after treatment with the formulation (Figure 5F).

**Figure 6:**
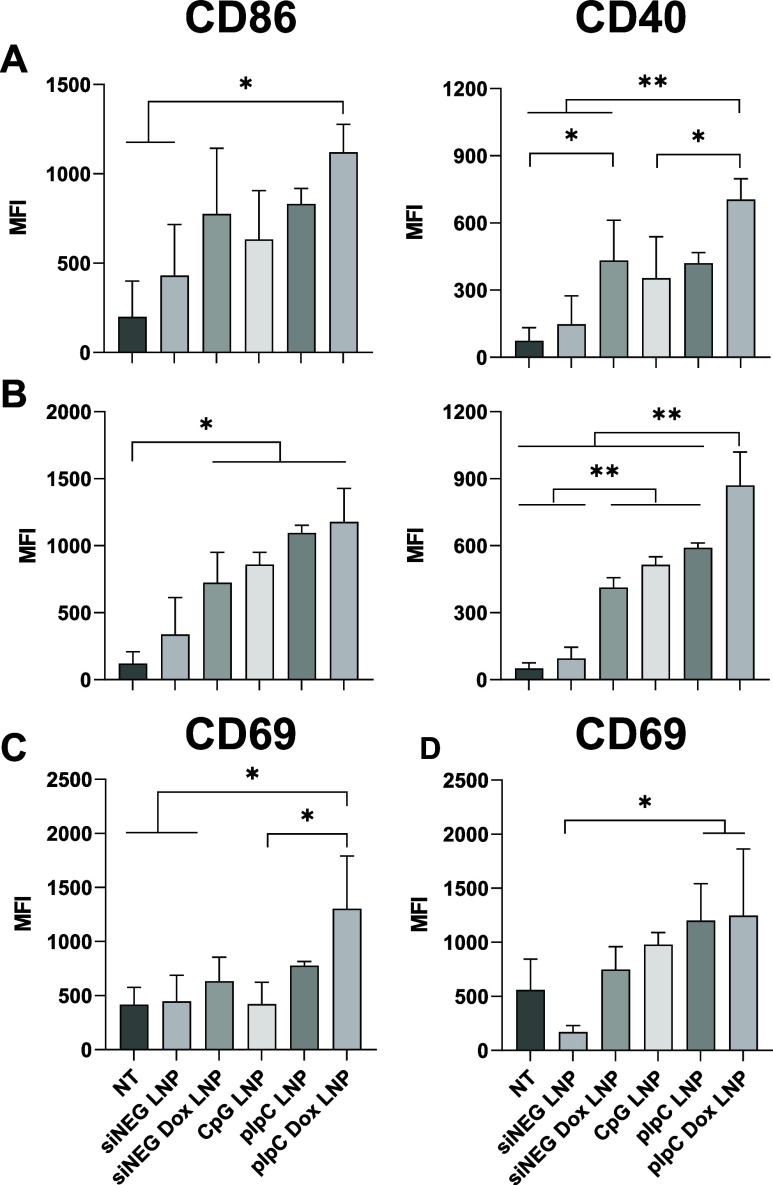
pIpC LNP and pIpC Dox LNP result in the activation of multiple leukocyte populations in vivo. GL261 brain tumor-bearing mice (C57BL/6 mice, *n* = 3 per group) were injected intracranially with LNP-formulated TLR agonists (pIpC LNP, CpG LNP) or a pIpC LNP incorporating Dox (pIpC LNP Dox) at a dose of 0.38 mg/kg TLR agonist and 1.18 mg/kg Dox on day 9 post tumor inoculation. As controls, LNPs incorporating siNEG (siNEG LNP) or siNEG plus Dox (siNEG Dox LNP) were used. A nontreated group (NT) served as a baseline. Mice were sacrificed 3 days post-treatment, and a single cell suspension was isolated from extracted tumors. Cells were stained for phenotypic markers of (A) DCs (CD45+, CD11b+, CD11c^high^) and (B) TAMs (CD45+, CD11b+) in addition to activation markers CD40 and CD86. For T cell activation, CD69 expression is shown for (C) all T cells (CD3+) or (D) CD8+ T cells (CD3+, CD8+). MFI on viable cells was assessed by FlowJo Star v 10.8 software. Data points represent mean and SD (*n* = 3 per group). Statistical analysis was carried out using one-way ANOVA, followed by the Tukey test with significance of ****p < 0.0001, ***p < 0.0002, **p < 0.005, and *p < 0.05.

In keeping with these findings, only the pIpC Dox LNP was able to increase the MFI of all activation markers on all immune cells (Figure 6). When looking at dendritic cells, all treatments apart from siNEG Dox and the negative control siNEG LNP caused an increase in the activation marker CD40 (Figure 6A). The CD86 increase on DC illustrates the efficiency of pIpC Dox LNP, as only this treatment group showed increased activation, with an average MFI of 1100 (control group MFI <200). The efficacy of pIpC Dox in activating immune cells was further highlighted by the increased CD40 MFI on TAMs, and this treatment showed a significant difference compared to all other treatments (Figure 6B). While for the in vitro studies, CD86 had shown the highest overall change in comparison to CD40, greater effects were seen for CD40 in the in vivo studies: CD86 increased approximately 5.5-fold, while CD40 showed an increase of nearly 10-fold when looking at pIpC Dox treatment in comparison to the control group. This effect was notably heightened on TAMs: CD40 MFI increased ∼20-fold, while CD86 increased ∼10-fold in TAMs in pIpC Dox treatment compared to the control group.

To investigate how the functionality of T cells changes following treatment, we chose the frequently used molecule CD69 as an activation marker. In line with the findings from TAMs and DCs, T cells equally showed upregulation after treatment with pIpC Dox LNP (Figure 6C). Unexpectedly, CD4+ T helper cells showed no significant difference, independent of the treatment (Figure S11). In contrast, in CD8+ cytotoxic T cells, CD69 MFI was increased in both the pIpC and pIpC Dox groups, with an average MFI of approximately 1200 and 1250, respectively, while the control group reached only an MFI of approximately 560 (Figure 6D).

**Figure 7:**
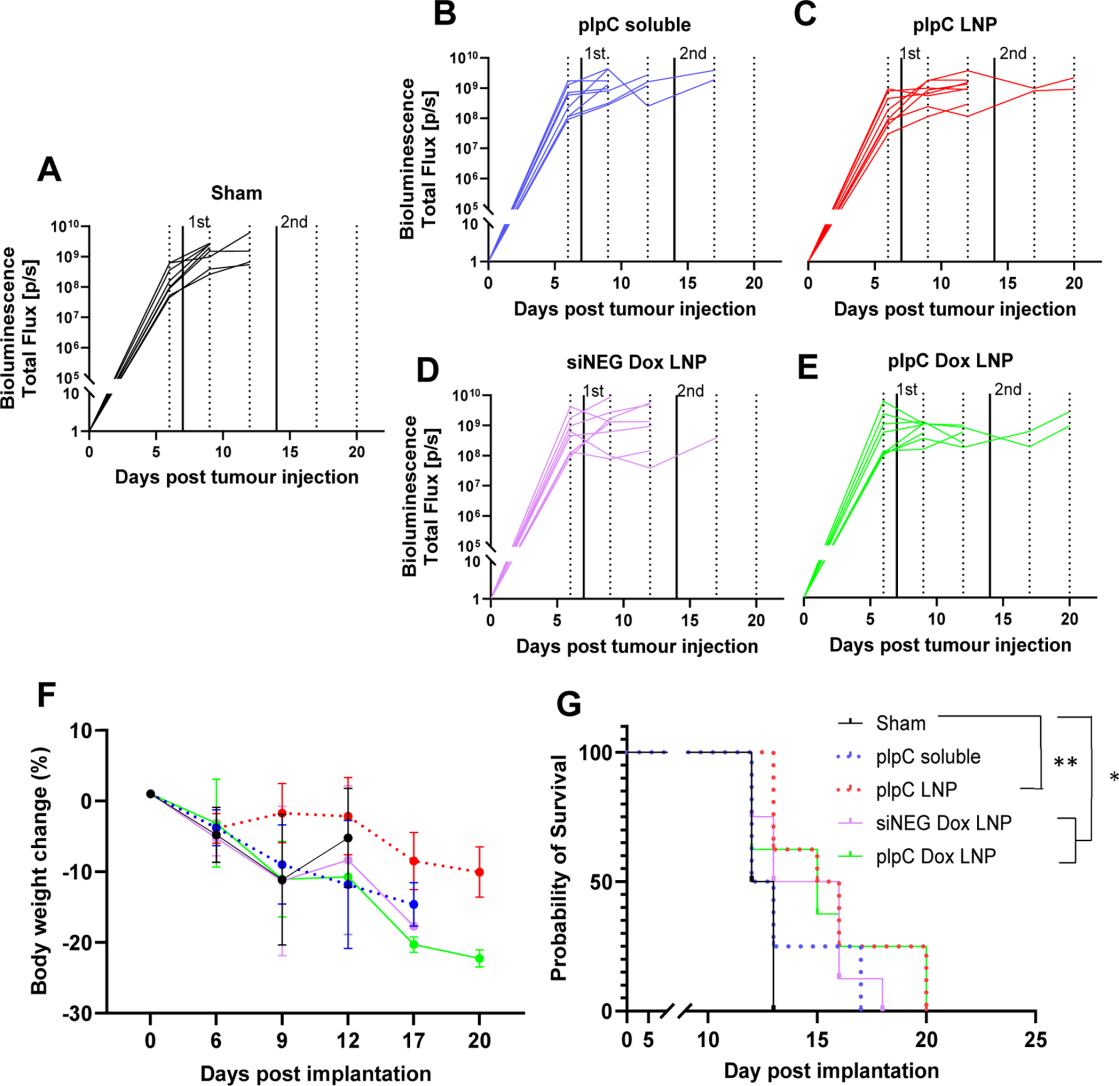
In vivo assessment of LNPs in the GL261 tumor model. Mice (C57BL/6 *n* = 8 per group) were implanted intracranially with 200 K GL261 cells. On days 7 and 14 (thick lines), mice were i.c. injected with either HEPES buffer (sham), soluble pIpC, pIpC LNP, siNEG Dox LNP, or pIpC Dox LNP. pIpC and DOX were used at a dosage of 450 and 1530 μg/kg, respectively. The tumor size was monitored for each mouse until the sacrifice of animals. D-luciferin was subcutaneously injected into animals of each group at a dosage of 150 mg/kg at multiple time intervals (dotted lines). The bioluminescence signal was recorded using IVIS immediately 10 min postinjection. The data are presented as a spaghetti plot for individual mice in each treatment group (A–E). (F) Changes in mouse weight relative to starting weight throughout the time course. (G) Kaplan–Meier survival curve of tumor-bearing mice following treatment. Statistical analysis was carried out using a Mantel–Cox test *p ≤ 0.033,**p ≤ 0.002.

A follow-up survival study was performed to establish the effect of the different therapeutics on mouse survival (Figures 7 and S12). Both plpC LNPs (15.5 days) and plpC Dox LNPs (15 days) showed significant improvement in survival compared to sham (12.5 days). Despite the significant improvement in immune cell infiltration observed in plpC Dox LNPs, this improvement did not translate to an improvement in survival compared to the plpC LNPs group. In the absence of plpC, siNEG Dox LNPs (14 days) also showed significant improvement in survival compared to sham (*p* < 0.05).

## 4 Discussion

In this study, the nucleic acid-based immune adjuvant pIpC and the ICD-inducing drug Dox were successfully coformulated in lipid nanoparticles and administered intracranially for the first time. The lipid nanoparticles display favorable physicochemical characteristics that showcase their suitability as a therapeutic delivery platform. Cytotoxicity studies revealed that the nucleic acid immune adjuvants, whether LNP-formulated or in their soluble form, only decreased immune cell viability by approximately 25%, even at the highest dosages tested (10 μg/mL), highlighting their potential to be administered safely without causing major cytotoxicity. The antitumoral effect of pIpC and CpG has been known for decades, and both have made it into clinical trials for GBM therapy.^35,36^ pIpC was tested in combination with radiotherapy as an intramuscular injection and demonstrated a survival benefit over historical studies without chemotherapy.^18^ CpG was likewise evaluated for its therapeutic potential and showed an improved overall survival when administered around the surgical cavity.^37^ Though the outcomes were generally positive, the studies raise questions about how immune adjuvant therapy can be further improved.

Formulation with lipids increases the hydrophobicity of the TLR agonists, which in turn facilitates cellular uptake and activation. A recent study tested bone marrow-derived dendritic cell and macrophage activation following treatment with pIpC and CpG in both soluble and liposomal form and found no clear superiority of either CpG or pIpC.^38^ In contrast with these findings, our study observed significant superiority of pIpC in activating different macrophage subtypes, DCs, and T cells. This may be due to the higher doses used in our study (10 vs 2 μg/mL). It may also be due to the differences in formulation, where Bayyurt et al. developed a liposome based on l-α-phosphatidylcholine and cholesterol, and we developed an LNP based on ionizable lipid. Ionizable lipid LNPs have surpassed liposomes in the nucleic acid delivery field due to their improved safety profile and also their efficient endosomal escape and subsequent cytosolic delivery of cargo. It may be possible to speculate that liposomes facilitate delivery to the endosome only, where TLR9 and TLR3 are expressed. LNPs, on the other hand, enable targeting of cytosolic RNA sensors, such as Mda-5, in addition to endosomal sensors, thus lending themselves to RNA-based adjuvant delivery.

While activating the innate immune system remains pivotal in the antitumoral immune response, previous clinical trials underscore the necessity for a multifaceted strategy to successfully eradicate GBM. In this respect, MHC class I emerges as a key player, as this molecule facilitates the crucial identification of cancer cells by CD8+ cytotoxic T cells. Given that one of the major mechanisms of immune escape in GBM is in fact the loss of MHC class I, reversing this effect should facilitate elimination.^39^ Several studies have shown that pIpC and its derivatives increase the expression of MHC class I on cancer cells.^34,40^ Interestingly, as Song, Han et al. have shown, Dox appears to be able to do so also.^33^ In keeping with the literature, our studies show increased median fluorescent intensity of MHC class I on GL261 brain cancer cells after incubation with pIpC LNP and pIpC Dox LNP, whereas neither siNEG Dox LNP nor CpG soluble or its LNP formulation was able to increase its expression significantly. It is likely that the absence of elevation using siNEG Dox LNP treatment is a result of the comparatively lower dosage of Dox used in this study.

After demonstrating that LNPs augment activation of immune cells as well as MHC class I expression on brain cancer cells in vitro, a coculture phagocytosis assay was performed to determine the dynamics between these two cell types. In keeping with the previous in vitro studies, the engulfment of GL261 cancer cells by J774 macrophages was enhanced after incubation with both pIpC LNP and pIpC Dox LNPs, independent of which cell type was treated. Interestingly, heightened phagocytosis was also observed when GL261 cancer cells and J774 macrophages were treated with pIpC-soluble and siNEG Dox LNP, respectively. These results suggest a common underlying mechanism through which both pIpC and Dox elicit pro-phagocytotic functions. Calreticulin could be responsible for this phenomenon, as it has been shown to be the pivotal factor in cellular uptake.^41^ In fact, both the anthracycline Dox as well as the TLR3 agonist pIpC have been shown to increase levels of the chemokine CXCL8, which in turn heightens calreticulin surface expression.^42,43^ Cancer cells seem to be specifically susceptible to anthracyclines, whereas macrophages have been shown to increase calreticulin expression after immune adjuvant treatment.^44^ Of particular significance is the consistent observation of the highest phagocytosis levels following pIpC Dox treatment across both treated cell lines. This pattern underpins the synergistic effect that both Dox and pIpC exert on cellular uptake. Future work might assess the underlying mechanisms that immune adjuvants and Dox elicit on calreticulin expression. Apart from increased calreticulin expression, the observation of highest phagocytosis upon pretreatment of J774 could also be explained by reprogramming into the M1 phenotype, as the previous results of this study have shown.

To test the formulations in vivo, we used an orthotopic GL261 GBM mouse model. In this study, the infiltration and activation of APC and T cells were assessed. Intratumoral pIpC Dox LNP administration proved to be the most effective treatment, significantly increasing the numbers of nearly all immune cell types except CD4+ T cells. Similar findings have been reported by Weng et al. in hepatocellular carcinoma in mouse models.^45^ These findings are mostly likely due to increased MHC class I expression, as a strong correlation with T cell infiltration has been found in melanoma.^46^ While the cause for the unchanged CD4+ T cell numbers remains unknown, the decreased CD4+/CD8+ T cell ratio holds immense significance. CD8+ T cells are also known as cytotoxic T cells, given their function of eradicating malignant or infected cells. Consequently, a decreased CD4+/CD8+ T cell ratio directly and strongly correlates with favorable clinical outcome.^47^ Another major component of the tumor microenvironment consists of tumor-associated macrophages. In fact, up to 30% of the TME consists of these TAMs.^48^ Historically, TAMs have been linked to tumor progression and poor outcomes in patients suffering from GBM.^49^ However, this perspective has proven to be oversimplified, as there are not only pro-tumorigenic M2 macrophages but also antitumorigenic M1 macrophages. Numerous studies have shown that it is feasible to reprogram TAMs into M1 macrophages, thereby inhibiting glioma growth.^50−52^ This study confirmed these previous findings, as pIpC LNP skews macrophages toward the M1 phenotype in vitro, independent of whether undifferentiated M0 macrophages were treated or differentiated M2 macrophages. Heightened levels of immune cells are only one part of the breakthrough that was achieved with pIpC Dox LNP combination therapy. As mentioned earlier, eradicating GBM also demands successful activation of immune cells, as several studies have found that a hallmark of GBM is T cell exclusion and exhaustion.^53^ Immunotherapy with pIpC Dox LNP overcomes this challenge, enabling the immune system to recognize and fight GBM. A pivotal factor for T cell activation is APC activation, which was observed in vitro upon immune adjuvant treatment as well as in vivo upon pIpC Dox LNP treatment. Once activated, APC display tumor-derived antigens as well as costimulatory molecules on their cell surface to T cells and secrete T cell chemotactic chemokines,^54^ enriching the tumor microenvironment with cytotoxic T cells. Despite the abundant evidence supporting the findings of this study and reinforcing the effectiveness of pIpC LNP treatment, an eminent absence of data exists concerning intracranial Dox treatment. The reason for this is simple: To date, there have been no studies evaluating the effects of Dox on brain tumors following intratumoral administration. Nevertheless, studies employing different modalities of delivery point toward a favorable outcome. As such, a recent study investigated the effects of Dox in combination with shockwaves that were used to disrupt the blood-brain barrier and found inhibition of tumor growth together with prolonged overall survival.^8^ The underlying mechanisms remained unknown, but this study suggests that increased activation of APC could be responsible for the findings. Another rationale for selecting pIpC over CpG for combination therapy with Dox in glioma stems from the observation that CpG can trigger the STAT3 signaling pathway, leading to the proliferation of glioma stem-like cells.^55^ These cells represent a significant obstacle in therapeutic efficacy and are currently under investigation as a focal point for research efforts.^56^ Despite the clear effectiveness of pIpC LNP and siNEG Dox LNP treatment, the highest immune cell infiltration and activation was observed upon pIpC Dox LNP combination therapy. Most interestingly, Dox and pIpC may exert some of their function through a common signaling pathway, as it has been shown that lack of TLR3 abrogated anthracycline response.^57^ Synergistically targeting the TLR3 receptor seems to be able to elicit the strongest immune cell response among all treatments.

The final study performed aimed to evaluate the long-term effects that resulted from the different treatments. Dox has been found to eradicate myeloid-derived suppressor cells and inhibit the function of remaining ones in a murine mammary cancer model.^58^ MDSCs cause downregulation of immune responses and have been studied as potential targets in GBM.^59^ The potential eradication and inhibition of MDSCs in combination with the observed activation and infiltration of immune cells may instigate an immune response of sufficient potency to autonomously eliminate GBM. In contrast to the changes in the tumor microenvironment, where pIpC Dox LNP showed superior changes, pIpC Dox LNP did not show improvement in mice survival over pIpC LNP or Dox LNP formulations. Future combinatory studies with other types of treatments, e.g., immunotherapies (immune checkpoint blockade or cancer vaccines) or temozolomide chemotherapy, will establish if Dox addition results in therapeutically relevant outcomes. Moving forward, the study’s focus should be on optimizing the dosage regimen of the pIpC LNP formulation, refining the frequency, timing, and concentration to maximize therapeutic benefits while minimizing toxicity. Additionally, combining this therapy with other modalities holds promise, such as the standard GBM treatment of temozolomide, immune checkpoint inhibitors (e.g., *anti*-PD-1 or *anti*-CTLA-4 antibodies), and cancer vaccines, to potentially enhance the immune response. Since doxorubicin did not significantly improve survival compared to pIpC alone, future studies could explore other immune-activating agents or chemotherapeutics that may work synergistically. Further preclinical testing in diverse GBM models, including patient-derived xenografts, will be crucial to assess the broader applicability. Ultimately, successful optimization would set the stage for translational studies in larger animal models and subsequent clinical trials in humans.

## 5 Conclusions

In conclusion, this study has shown that encapsulation of plpC into LNP offers an advantage compared to soluble plpC such as increased MHC class I expression on the cancer cells, enhanced DCs, M0, M1, and M2 activation, and M2 to M1 macrophage reprogramming leading to efficient clearance of cancer cells by the activated macrophages in vitro. The addition of Dox into plpC LNP showed extended benefits such as enhanced DCs, TAM, and CD8 cell infiltrations, in their active form, into the orthotopically implanted GBM compared to plpC LNP. Dox LNP, pIpC LNP, and pIpC Dox LNP offered improved survival profiles in vivo. This study is another showcase of the advantages LNPs can offer for GBM immunotherapy and chemotherapy as standalone treatments or in combination with other therapeutics in the future.

## Supporting Information Available

Details of LNP characterization, in vitro behavior of LNPs, flow cytometric gating strategies, and addition in vivo data (PDF)

## References

[ref1] Davis M. E. Glioblastoma: overview of disease and treatment. Clin. J. Oncol. Nurs. 2016, 20 (5), S2–S8. 10.1188/16.CJON.S1.2-8. PMC5123811 27668386

[ref2] Stupp R.; Hegi M. E.; Mason W. P.; Van Den Bent M. J.; Taphoorn M. J.; Janzer R. C.; Ludwin S. K.; Allgeier A.; Fisher B.; Belanger K.; et al. Effects of radiotherapy with concomitant and adjuvant Temozolomide versus radiotherapy alone on survival in glioblastoma in a randomised phase III study: 5-year analysis of the EORTC-NCIC trial. Lancet Oncol. 2009, 10 (5), 459–466. 10.1016/S1470-2045(09)70025-7. 19269895

[ref3] Zhou J.; Wang G.; Chen Y.; Wang H.; Hua Y.; Cai Z. Immunogenic cell death in cancer therapy: Present and emerging inducers. J. Cell. Mol. Med. 2019, 23 (8), 4854–4865. 10.1111/jcmm.14356. 31210425 PMC6653385

[ref4] Hodge J. W.; Garnett C. T.; Farsaci B.; Palena C.; Tsang K. Y.; Ferrone S.; Gameiro S. R. Chemotherapy-induced immunogenic modulation of tumor cells enhances killing by cytotoxic T lymphocytes and is distinct from immunogenic cell death. Int. J. Cancer 2013, 133 (3), 624–636. 10.1002/ijc.28070. 23364915 PMC3663913

[ref5] Huang K. C.-Y.; Chiang S.-F.; Yang P.-C.; Ke T.-W.; Chen T.-W.; Hu C.-H.; Huang Y. W.; Chang H. Y.; Chen W. T. L.; Chao K. S. C. Immunogenic cell death by the novel topoisomerase i inhibitor tlc388 enhances the therapeutic efficacy of radiotherapy. Cancers 2021, 13 (6), 1218 10.3390/cancers13061218. 33799527 PMC7998596

[ref6] Krysko D. V.; Garg A. D.; Kaczmarek A.; Krysko O.; Agostinis P.; Vandenabeele P. Immunogenic cell death and DAMPs in cancer therapy. Nat. Rev. Cancer 2012, 12 (12), 860–875. 10.1038/nrc3380. 23151605

[ref7] Fabian K. P.; Wolfson B.; Hodge J. W. From immunogenic cell death to immunogenic modulation: select chemotherapy regimens induce a spectrum of immune-enhancing activities in the tumor microenvironment. Front. Oncol. 2021, 11, 728018 10.3389/fonc.2021.728018. 34497771 PMC8419351

[ref8] Liao W.-H.; Hsiao M.-Y.; Kung Y.; Huang A. P.-H.; Chen W.-S. Investigation of the therapeutic effect of doxorubicin combined with focused shockwave on glioblastoma. Front. Oncol. 2021, 11, 2977 10.3389/fonc.2021.711088. PMC8356050 34395286

[ref9] Hau P.; Fabel K.; Baumgart U.; Rümmele P.; Grauer O.; Bock A.; Dietmaier C.; Dietmaier W.; Dietrich J.; Dudel C.; et al. Pegylated liposomal doxorubicin-efficacy in patients with recurrent high-grade glioma. Cancer 2004, 100 (6), 1199–1207. 10.1002/cncr.20073. 15022287

[ref10] Himes B. T.; Geiger P. A.; Ayasoufi K.; Bhargav A. G.; Brown D. A.; Parney I. F. Immunosuppression in Glioblastoma: Current Understanding and Therapeutic Implications. Front. Oncol. 2021, 11, 770561 10.3389/fonc.2021.770561. 34778089 PMC8581618

[ref11] Chen X.; Zhang Y.; Fu Y. The Critical Role of Toll-like Receptor-mediated Signaling in Cancer Immunotherapy. Med. Drug Discovery 2022, 14, 100122 10.1016/j.medidd.2022.100122.

[ref12] Wang Q.-T.; Nie Y.; Sun S.-N.; Lin T.; Han R.-J.; Jiang J.; Li Z.; Li J.-Q.; Xiao Y.-P.; Fan Y.-Y.; et al. Tumor-associated antigen-based personalized dendritic cell vaccine in solid tumor patients. Cancer Immunol., Immunother. 2020, 69 (7), 1375–1387. 10.1007/s00262-020-02496-w. 32078016 PMC11027674

[ref13] Migliorini D.; Dutoit V.; Allard M.; Grandjean Hallez N.; Marinari E.; Widmer V.; Philippin G.; Corlazzoli F.; Gustave R.; Kreutzfeldt M.; et al. Phase I/II trial testing safety and immunogenicity of the multipeptide IMA950/poly-ICLC vaccine in newly diagnosed adult malignant astrocytoma patients. Neurooncology 2019, 21 (7), 923–933. 10.1093/neuonc/noz040. PMC6620642 30753611

[ref14] Okada H.; Kalinski P.; Ueda R.; Hoji A.; Kohanbash G.; Donegan T. E.; Mintz A. H.; Engh J. A.; Bartlett D. L.; Brown C. K.; et al. Induction of CD8+ T-cell responses against novel glioma–associated antigen peptides and clinical activity by vaccinations with α-type 1 polarized dendritic cells and polyinosinic-polycytidylic acid stabilized by lysine and carboxymethylcellulose in patients with recurrent malignant glioma. J. Clin. Oncol. 2011, 29 (3), 330–336. 10.1200/JCO.2010.30.7744. 21149657 PMC3056467

[ref15] Grauer O. M.; Molling J. W.; Bennink E.; Toonen L. W.; Sutmuller R. P.; Nierkens S.; Adema G. J. TLR ligands in the local treatment of established intracerebral murine gliomas. J. Immunol. 2008, 181 (10), 6720–6729. 10.4049/jimmunol.181.10.6720. 18981089

[ref16] Mac’Cheever M. A. Twelve immunotherapy drugs that could cure cancers. Immunol. Rev. 2008, 222 (1), 357–368. 10.1111/j.1600-065x.2008.00604.x. 18364014

[ref17] Shir A.; Ogris M.; Wagner E.; Levitzki A. Correction: EGF receptor-targeted synthetic double-stranded RNA eliminates glioblastoma, breast cancer, and adenocarcinoma tumors in mice. PLoS Med. 2007, 4 (8), e266 10.1371/journal.pmed.0040266. PMC1298941 16318410

[ref18] Butowski N.; Chang S. M.; Junck L.; DeAngelis L. M.; Abrey L.; Fink K.; Cloughesy T.; Lamborn K. R.; Salazar A. M.; Prados M. D. A phase II clinical trial of poly-ICLC with radiation for adult patients with newly diagnosed supratentorial glioblastoma: a North American Brain Tumor Consortium (NABTC01–05). J. Neuro-Oncol. 2009, 91 (2), 175–182. 10.1007/s11060-008-9693-3. PMC4779120 18797818

[ref19] Carpentier A.; Laigle-Donadey F.; Zohar S.; Capelle L.; Behin A.; Tibi A.; Martin-Duverneuil N.; Sanson M.; Lacomblez L.; Taillibert S.; et al. Phase 1 trial of a CpG oligodeoxynucleotide for patients with recurrent glioblastoma. Neurooncology 2006, 8 (1), 60–66. 10.1215/S1522851705000475. PMC1871923 16443949

[ref20] Ruan S.; Zhou Y.; Jiang X.; Gao H. Rethinking CRITID procedure of brain targeting drug delivery: circulation, blood brain barrier recognition, intracellular transport, diseased cell targeting, internalization, and drug release. Advanced Science 2021, 8 (9), 2004025 10.1002/advs.202004025. 33977060 PMC8097396

[ref21] El Maghraby G. M.; Arafa M. F. Liposomes for enhanced cellular uptake of anticancer agents. Curr. Drug Delivery 2020, 17 (10), 861–873. 10.2174/1567201817666200708113131. 32640957

[ref22] Miller M.; Zheng Y.; Gadde S.; Pfirschke C.; Zope H.; Engblom C.; Kohler R. H.; Iwamoto Y.; Yang K. S.; Askevold B.; et al. Tumour-associated macrophages act as a slow-release reservoir of nano-therapeutic Pt (IV) pro-drug. Nat. Commun. 2015, 6, 8692 10.1038/ncomms9692. 26503691 PMC4711745

[ref23] Agier J.; Żelechowska P.; Kozłowska E.; Brzezińska-Błaszczyk E. Expression of surface and intracellular Toll-like receptors by mature mast cells. Cent. Eur. J. Immunol. 2016, 41 (4), 333–338. 10.5114/ceji.2016.65131. 28450795 PMC5382879

[ref24] Xu L.; Zhang W.; Park H.-B.; Kwak M.; Oh J.; Lee P. C.; Jin J. O. Indocyanine green and poly I: C containing thermo-responsive liposomes used in immune-photothermal therapy prevent cancer growth and metastasis. J. ImmunoTher. Cancer. 2019, 7 (1), 220 10.1186/s40425-019-0702-1. 31412934 PMC6694491

[ref25] Colapicchioni V.; Palchetti S.; Pozzi D.; Marini E. S.; Riccioli A.; Ziparo E.; Papi M.; Amenitsch H.; Caracciolo G. Killing cancer cells using nanotechnology: novel poly (I: C) loaded liposome–silica hybrid nanoparticles. J. Mater. Chem. B 2015, 3 (37), 7408–7416. 10.1039/C5TB01383F. 32262767

[ref26] Hammarlund-Udenaes M.; Fridén M.; Syvänen S.; Gupta A. On the rate and extent of drug delivery to the brain. Pharm. Res. 2008, 25, 1737–1750. 10.1007/s11095-007-9502-2. 18058202 PMC2469271

[ref27] de Lange E. C.; Danhof M. Considerations in the use of cerebrospinal fluid pharmacokinetics to predict brain target concentrations in the clinical setting: implications of the barriers between blood and brain. Clin. Pharmacokinet. 2002, 41, 691–703. 10.2165/00003088-200241100-00001. 12162757

[ref28] Walters A. A.; Santacana-Font G.; Li J.; Routabi N.; Qin Y.; Claes N.; Bals S.; Tzu-Wen Wang J.; Al-Jamal K. T. Nanoparticle-mediated in situ molecular reprogramming of immune checkpoint interactions for cancer immunotherapy. ACS Nano 2021, 15 (11), 17549–17564. 10.1021/acsnano.1c04456. 34677938 PMC8613910

[ref29] Mayer L. D.; Cullis P. R.; Bally M. B. The use of transmembrane pH gradient-driven drug encapsulation in the pharmacodynamic evaluation of liposomal doxorubicin. J. Liposome Res. 1994, 4 (1), 529–553. 10.3109/08982109409037060.

[ref30] Cao Y.; Ding S.; Zeng L.; Miao J.; Wang K.; Chen G.; Li C.; Zhou J.; Bian X. w.; Tian G. Reeducating tumor-associated macrophages using CpG@ Au nanocomposites to modulate immunosuppressive microenvironment for improved radio-immunotherapy. ACS Appl. Mater. Interfaces 2021, 13 (45), 53504–53518. 10.1021/acsami.1c07626. 34704726

[ref31] Dacoba T. G.; Anfray C.; Mainini F.; Allavena P.; Alonso M. J.; Torres Andón F.; Crecente-Campo J. Arginine-based Poly (I: C)-loaded nanocomplexes for the polarization of macrophages toward M1-antitumoral effectors. Front. Immunol. 2020, 11, 1412 10.3389/fimmu.2020.01412. 32733469 PMC7358452

[ref32] Burster T.; Gärtner F.; Bulach C.; Zhanapiya A.; Gihring A.; Knippschild U. Regulation of MHC I molecules in glioblastoma cells and the sensitizing of NK cells. Pharmaceuticals 2021, 14 (3), 236 10.3390/ph14030236. 33800301 PMC7998501

[ref33] Song P.; Han X.; Zheng R.; Yan J.; Wu X.; Wang Y.; Zhang H. Upregulation of MHC-I and downregulation of PD-L1 expression by doxorubicin and deferasirox codelivered liposomal nanoparticles for chemoimmunotherapy of melanoma. Int. J. Pharm. 2022, 624, 122002 10.1016/j.ijpharm.2022.122002. 35817272

[ref34] Zhu X.; Nishimura F.; Sasaki K.; Fujita M.; Dusak J. E.; Eguchi J.; Fellows-Mayle W.; Storkus W. J.; Walker P. R.; Salazar A. M.; et al. Toll like receptor-3 ligand poly-ICLC promotes the efficacy of peripheral vaccinations with tumor antigen-derived peptide epitopes in murine CNS tumor models. J. Transl. Med. 2007, 5, 1–15. 10.1186/1479-5876-5-10. 17295916 PMC1802742

[ref35] Fitzgerald K. A.; Kagan J. C. Toll-like receptors and the control of immunity. Cell 2020, 180 (6), 1044–1066. 10.1016/j.cell.2020.02.041. 32164908 PMC9358771

[ref36] Shime H.; Matsumoto M.; Oshiumi H.; Tanaka S.; Nakane A.; Iwakura Y.; Tahara H.; Inoue N.; Seya T. Toll-like receptor 3 signaling converts tumor-supporting myeloid cells to tumoricidal effectors. Proc. Natl. Acad. Sci. U.S.A. 2012, 109 (6), 2066–2071. 10.1073/pnas.1113099109. 22308357 PMC3277567

[ref37] Ursu R.; Carpentier A.; Metellus P.; Lubrano V.; Laigle-Donadey F.; Capelle L.; Guyotat J.; Langlois O.; Bauchet L.; Desseaux K.; et al. Intracerebral injection of CpG oligonucleotide for patients with de novo glioblastoma—a phase II multicentric, randomised study. Eur. J. Cancer 2017, 73, 30–37. 10.1016/j.ejca.2016.12.003. 28142059

[ref38] Bayyurt B.; Tincer G.; Almacioglu K.; Alpdundar E.; Gursel M.; Gursel I. Encapsulation of two different TLR ligands into liposomes confer protective immunity and prevent tumor development. J. Controlled Release 2017, 247, 134–144. 10.1016/j.jconrel.2017.01.004. 28069554

[ref39] Dhatchinamoorthy K.; Colbert J. D.; Rock K. L. Cancer immune evasion through loss of MHC class I antigen presentation. Front. Immunol. 2021, 12, 636568 10.3389/fimmu.2021.636568. 33767702 PMC7986854

[ref40] Kalbasi A.; Tariveranmoshabad M.; Hakimi K.; Kremer S.; Campbell K. M.; Funes J. M.; Vega-Crespo A.; Parisi G.; Champekar A.; Nguyen C.; et al. Uncoupling interferon signaling and antigen presentation to overcome immunotherapy resistance due to JAK1 loss in melanoma. Sci. Transl. Med. 2020, 12 (565), eabb0152 10.1126/scitranslmed.abb0152. 33055240 PMC8053376

[ref41] Chao M. P.; Jaiswal S.; Weissman-Tsukamoto R.; Alizadeh A. A.; Gentles A. J.; Volkmer J.; Weiskopf K.; Willingham S. B.; Raveh T.; Park C. Y.; et al. Calreticulin is the dominant pro-phagocytic signal on multiple human cancers and is counterbalanced by CD47. Sci. Transl. Med. 2010, 2 (63), 63ra94 10.1126/scitranslmed.3001375. PMC4126904 21178137

[ref42] Sukkurwala A.; Martins I.; Wang Y.; Schlemmer F.; Ruckenstuhl C.; Durchschlag M.; Michaud M.; Senovilla L.; Sistigu A.; Ma Y.; et al. Immunogenic calreticulin exposure occurs through a phylogenetically conserved stress pathway involving the chemokine CXCL8. Cell Death Differ. 2014, 21 (1), 59–68. 10.1038/cdd.2013.73. 23787997 PMC3857625

[ref43] Grimstad Ø.; Pukstad B.; Stenvik J.; Espevik T. Oligodeoxynucleotides inhibit Toll-like receptor 3 mediated cytotoxicity and CXCL8 release in keratinocytes. Exp. Dermatol. 2012, 21 (1), 7–12. 10.1111/j.1600-0625.2011.01390.x. 22082188

[ref44] Feng M.; Chen J. Y.; Weissman-Tsukamoto R.; Volkmer J.-P.; Ho P. Y.; McKenna K. M.; Cheshier S.; Zhang M.; Guo N.; Gip P.; et al. Macrophages eat cancer cells using their own calreticulin as a guide: roles of TLR and Btk. Proc. Natl. Acad. Sci. U.S.A. 2015, 112 (7), 2145–2150. 10.1073/pnas.1424907112. 25646432 PMC4343163

[ref45] Weng M.-T.; Yang S.-F.; Liu S.-Y.; Hsu Y.-C.; Wu M.-C.; Chou H.-C.; Chiou L. L.; Liang J. D.; Wang L. F.; Lee H. S.; et al. In situ vaccination followed by intramuscular poly-ICLC injections for the treatment of hepatocellular carcinoma in mouse models. Pharmacol. Res. 2023, 188, 106646 10.1016/j.phrs.2023.106646. 36621619

[ref46] Al-Batran S.-E.; Rafiyan M.-R.; Atmaca A.; Neumann A.; Karbach J.; Bender A.; Weidmann E.; Altmannsberger H. M.; Knuth A.; Jäger E. Intratumoral T-cell infiltrates and MHC class I expression in patients with stage IV melanoma. Cancer Res. 2005, 65 (9), 3937–3941. 10.1158/0008-5472.CAN-04-4621. 15867394

[ref47] Han S.; Zhang C.; Li Q.; Dong J.; Liu Y.; Huang Y.; Jiang T.; Wu A. Tumour-infiltrating CD4+ and CD8+ lymphocytes as predictors of clinical outcome in glioma. Br. J. Cancer 2014, 110 (10), 2560–2568. 10.1038/bjc.2014.162. 24691423 PMC4021514

[ref48] Hambardzumyan D.; Gutmann D. H.; Kettenmann H. The role of microglia and macrophages in glioma maintenance and progression. Nat. Neurosci. 2016, 19 (1), 20–27. 10.1038/nn.4185. 26713745 PMC4876023

[ref49] Sørensen M.; Dahlrot R.; Boldt H.; Hansen S.; Kristensen B. Tumour-associated microglia/macrophages predict poor prognosis in high-grade gliomas and correlate with an aggressive tumour subtype. Neuropathol. Appl. Neurobiol. 2018, 44 (2), 185–206. 10.1111/nan.12428. 28767130

[ref50] Pyonteck S. M.; Akkari L.; Schuhmacher A. J.; Bowman R. L.; Sevenich L.; Quail D. F.; Olson O. C.; Quick M. L.; Huse J. T.; Teijeiro V.; et al. CSF-1R inhibition alters macrophage polarization and blocks glioma progression. Nat. Med. 2013, 19 (10), 1264–1272. 10.1038/nm.3337. 24056773 PMC3840724

[ref51] Zhang L.; Alizadeh D.; Van Handel M.; Kortylewski M.; Yu H.; Badie B. Stat3 inhibition activates tumor macrophages and abrogates glioma growth in mice. Glia 2009, 57 (13), 1458–1467. 10.1002/glia.20863. 19306372

[ref52] Fujiwara Y.; Komohara Y.; Kudo R.; Tsurushima K.; Ohnishi K.; Ikeda T.; et al. Oleanolic acid inhibits macrophage differentiation into the M2 phenotype and glioblastoma cell proliferation by suppressing the activation of STAT3. Oncol. Rep. 2011, 26 (6), 1533–1537. 10.3892/or.2011.1454. 21922144

[ref53] Mohme M.; Schliffke S.; Maire C. L.; Rünger A.; Glau L.; Mende K. C.; Matschke J.; Gehbauer C.; Akyüz N.; Zapf S.; et al. Immunophenotyping of Newly Diagnosed and Recurrent Glioblastoma Defines Distinct Immune Exhaustion Profiles in Peripheral and Tumor-infiltrating Lymphocytes. Clin. Cancer Res. 2018, 24 (17), 4187–4200. 10.1158/1078-0432.CCR-17-2617. 29444930

[ref54] Alfei F.; Ho P.-C.; Lo W.-L. DCision-making in tumors governs T cell anti-tumor immunity. Oncogene 2021, 40 (34), 5253–5261. 10.1038/s41388-021-01946-8. 34290401 PMC8390370

[ref55] Fehri E.; Ennaifer E.; Bel Haj Rhouma R.; Ardhaoui M.; Boubaker S. TLR9 and Glioma: Friends or Foes?. Cells 2023, 12 (1), 152 10.3390/cells12010152. PMC9818384 36611945

[ref56] Yi Y.; Hsieh I.-Y.; Huang X.; Li J.; Zhao W. Glioblastoma stem-like cells: characteristics, microenvironment, and therapy. Front. Pharmacol. 2016, 7, 477 10.3389/fphar.2016.00477. 28003805 PMC5141588

[ref57] Sistigu A.; Yamazaki T.; Vacchelli E.; Chaba K.; Enot D. P.; Adam J.; Vitale I.; Goubar A.; Baracco E. E.; Remédios C.; et al. Cancer cell–autonomous contribution of type I interferon signaling to the efficacy of chemotherapy. Nat. Med. 2014, 20 (11), 1301–1309. 10.1038/nm.3708. 25344738

[ref58] Alizadeh D.; Trad M.; Hanke N. T.; Larmonier C. B.; Janikashvili N.; Bonnotte B.; Katsanis E.; Larmonier N. Doxorubicin eliminates myeloid-derived suppressor cells and enhances the efficacy of adoptive T-cell transfer in breast cancer. Cancer Res. 2014, 74 (1), 104–118. 10.1158/0008-5472.CAN-13-1545. 24197130 PMC3896092

[ref59] Alban T. J.; Bayik D.; Otvos B.; Rabljenovic A.; Leng L.; Jia-Shiun L.; Roversi G.; Lauko A.; Momin A. A.; Mohammadi A. M.; et al. Glioblastoma myeloid-derived suppressor cell subsets express differential macrophage migration inhibitory factor receptor profiles that can be targeted to reduce immune suppression. Front. Immunol. 2020, 11, 1191 10.3389/fimmu.2020.01191. 32625208 PMC7315581

