# Supplementary material for: Polyinosinic/Polycytidylic Lipid Nanoparticles Enhance Immune Cell Infiltration and Improve Survival in the Glioblastoma Mouse Model
Source: Mol Pharm. 2024 Nov 18;21(12):6339–52. doi: 10.1021/acs.molpharmaceut.4c00875 (PMC11615939; doi:10.1021/acs.molpharmaceut.4c00875)
Supplement: Supplementary file 1 — mp4c00875_si_001.pdf [file mp4c00875_si_001.pdf]

Polyinosinic:polycytidylic Lipid Nanoparticles Enhance Immune Cell infiltration and Improves Survival in Glioblastoma Mouse Model

Melanie MT Brüßeler [1,2,#], Alaa Zam[1,#], Víctor Manuel Moreno Zafra [1], Nadia Rouatbi[1], Osama WM Hassuneh[1], Alessia Marrocu[1], Revadee Liam-Or[1,3] Hend Mohamed Abdel-Bar[1,4], Adam Alexander Walters[1,#], and Khuloud T. Al-Jamal[1,3,*]

1. Institute of Pharmaceutical Science, King's College London, Franklin-Wilkins Building, 150 Stamford Street, London SE1 9NH, UK
2. Ludwig Maximilians University, Munich, München, Bayern, Germany, 80539
3. Department of Pharmacology and Pharmacy, Li Ka Shing Faculty of Medicine, The University of Hong Kong, Hong Kong Special Administrative Region, China
4. Department of Pharmaceutics, Faculty of Pharmacy, University of Sadat City, P.O. box: 32958 Egypt

[#] Equal contribution to the work

* Corresponding authors

E-mail: khuloud.al-jamal@kcl.ac.uk

Keywords: Doxorubicin, GL261, pIpC, CpG, cancer, combinatory.

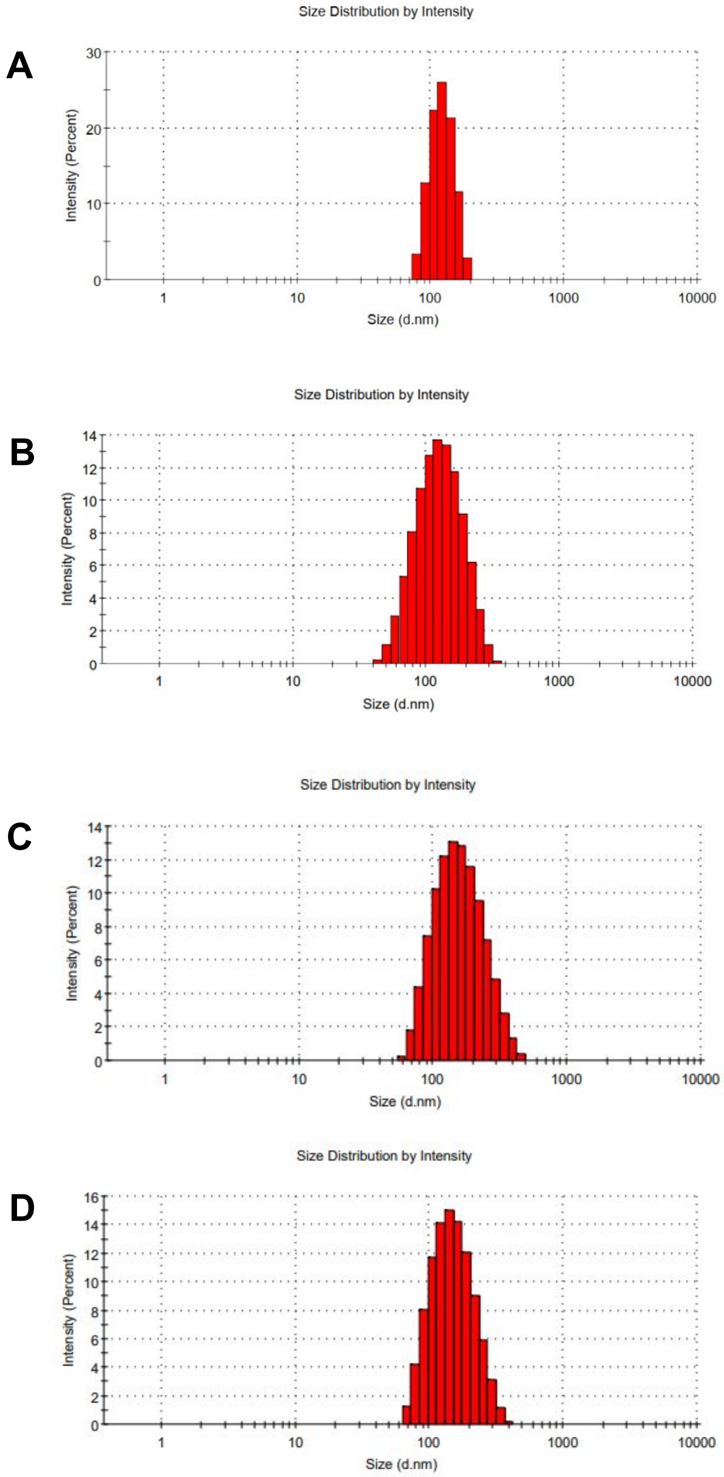

Figure S1. Histograms displaying LNP size. (A) pIpC LNP, **(B)** pIpC Dox, **(C)** CpG LNP and **(D)** CpG Dox LNP. LNP were diluted 10 times with PBS prior to analysis with Nanosizer ZS Series (Malvern Instruments, MA). All measurements were carried out as triplicate.

A

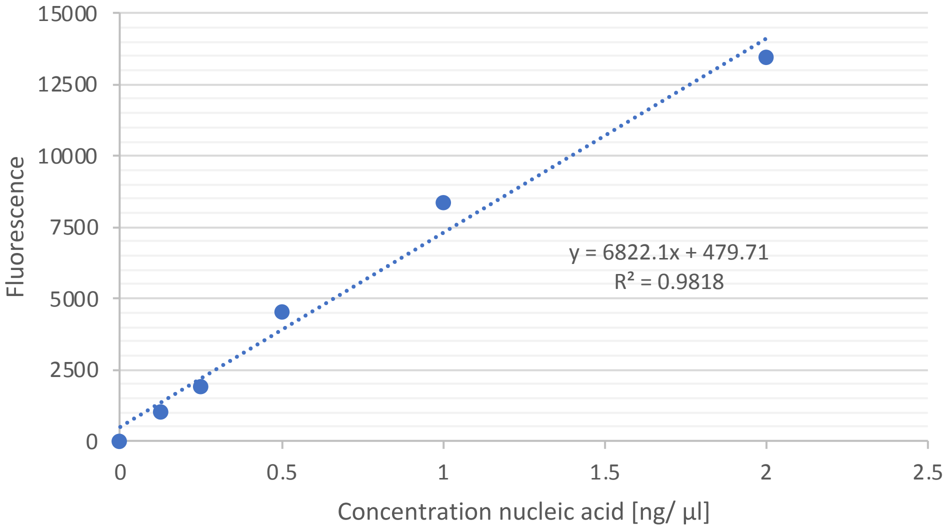

B

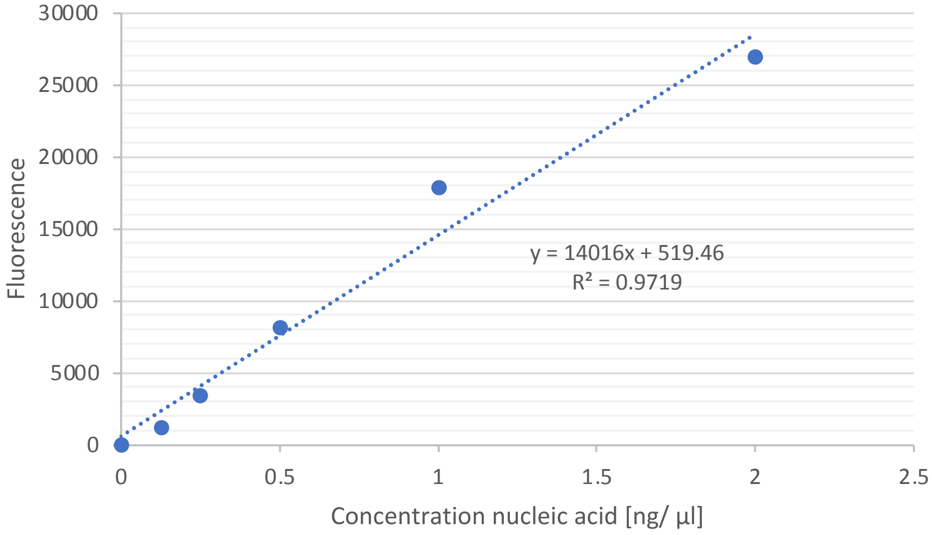

Figure S2. Representative standard curve for calculation of nucleic acid encapsulation efficiency Ribogreen assay was performed with a standard curve of mRNA in PBS in the presence or absence of 1% Triton X-100 ((**A**) and (**B**) respectively). A serial dilution ranging from 2.5 to – ng/ µl was made with either soluble pIpC or CpG. The coefficient of determination (R^2) was always above 0.95 for all standard curves.

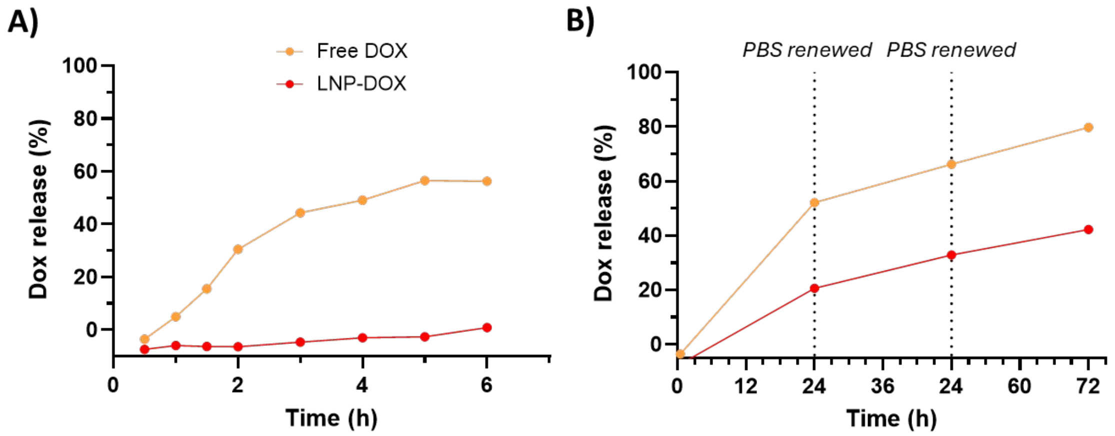

Figure S3. Release profile of Dox in free (Free-DOX) and encapsulated (LNP-DOX) form at short term (6 h) and mid-term (72 h) times. Both samples (43.4 mg Dox in 1 mL PBS) were placed in a dialysis cassette (3.5 kDa) and dialyzed against 15 mL of PBS 1X (pH 7.4) under incubation at 37 °C and 250 rpm. Aliquots of 0.2 mL were taken each time point and the same volume of PBS was added (**A**). For mid-term assay, the PBS dialysis solution was renewed every 24 h (**B**). Dox was detected by fluorometry with excitation and emission wavelengths of 485 and 590 nm, respectively.

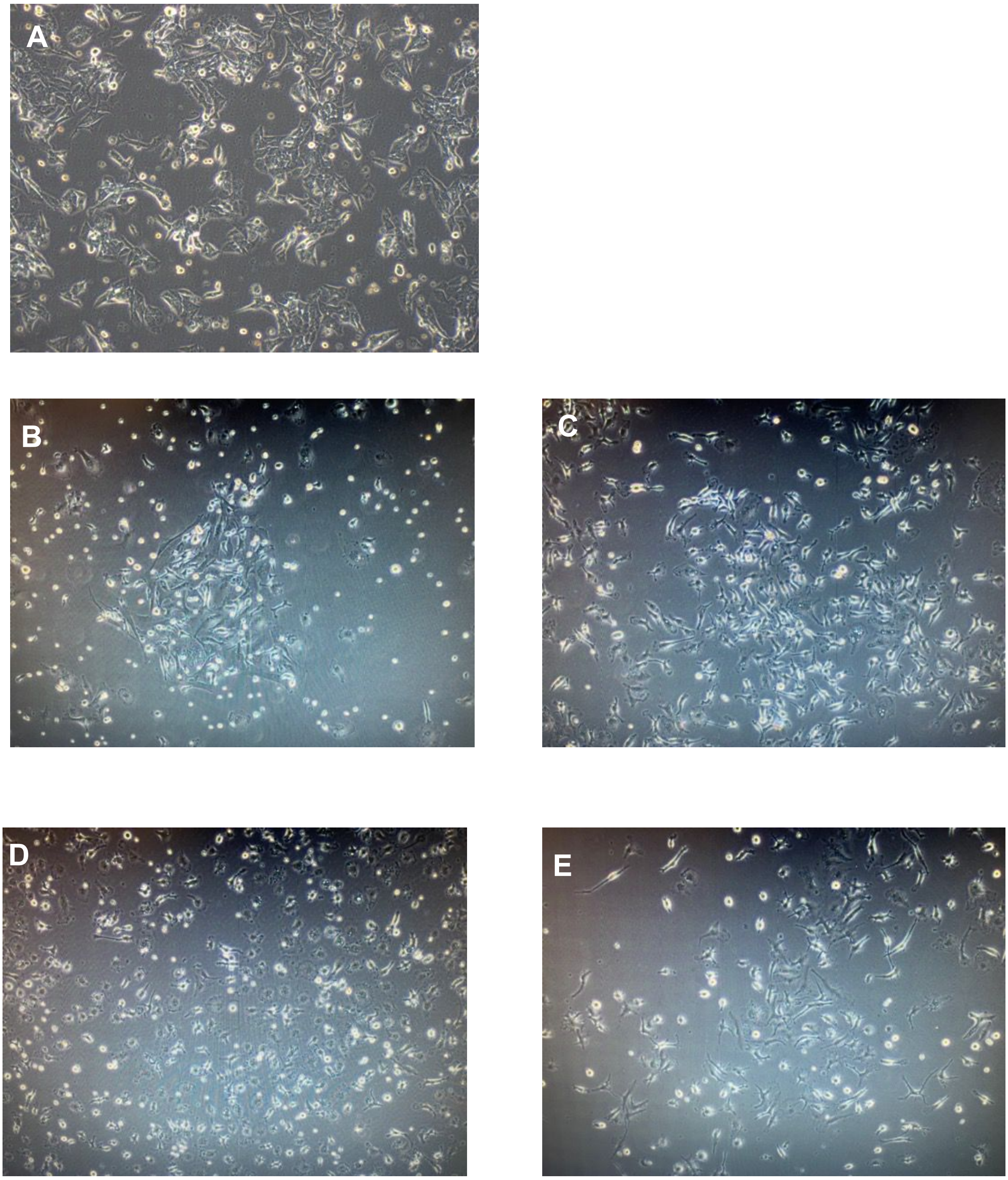

Figure S4. Photo-microscopic images of cancer and immune cells. (A) GL261 glioblastoma, **(B)** DCs, **(C)** M0, **(D)** M1 and **(E)** M2 Macrophages. Photos were taken with an Olympus® inverted microscope and Micropublisher 3 camera at 10x4 magnification.

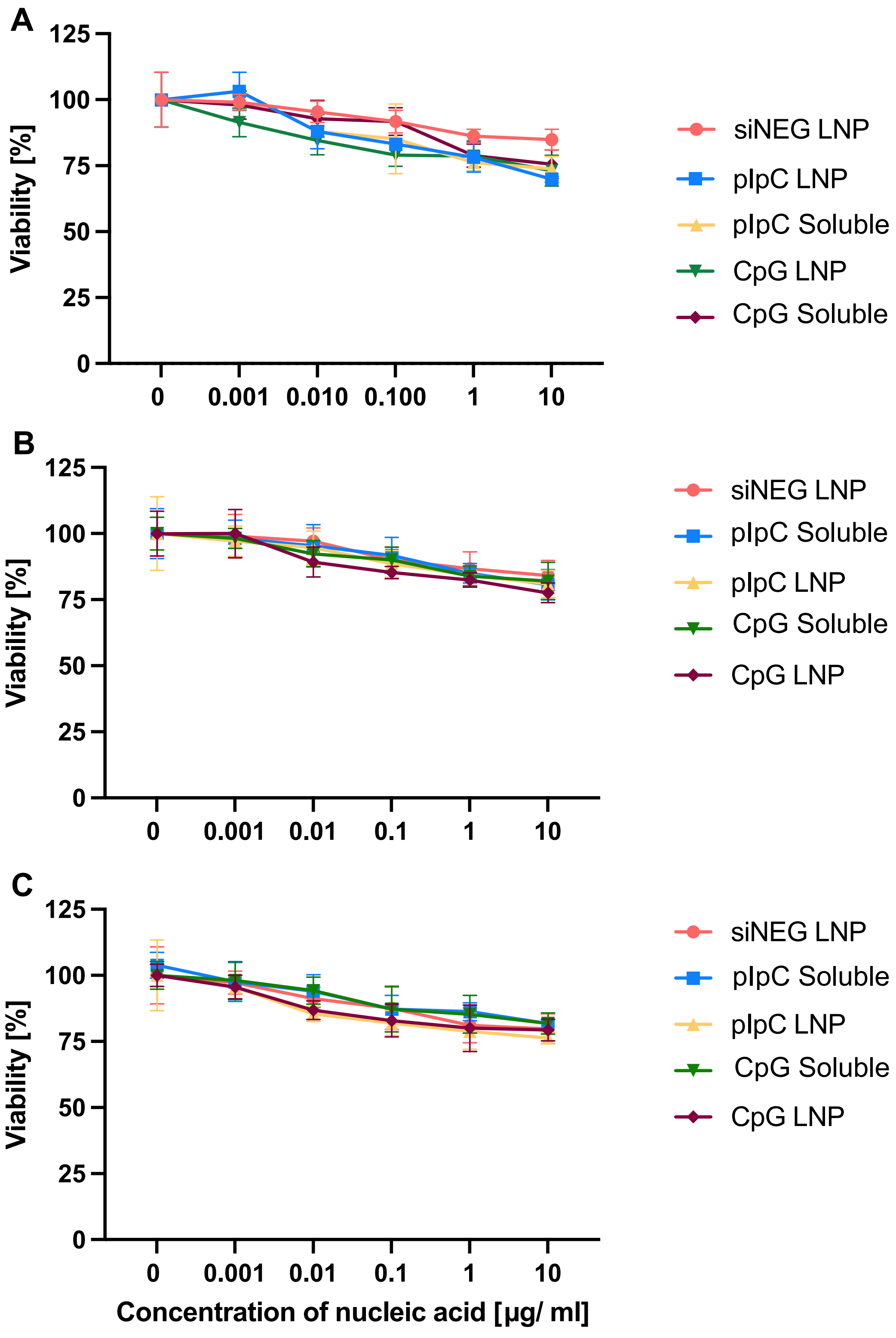

Figure S5. Cytotoxicity of nucleic acid LNPs on murine glioblastoma cancer cells antigen presenting cells *in vitro*. Gl261.luc cells (**A**) were seeded at 5k/ 100µl, DC (**B**) and M0 Macrophages (**C**) at 750k/ 100µ in a 96 well plate. After 24h incubation, cells were incubated with different nucleic acid formulations at increasing concentrations for 48h. Cell viability was assessed *via* MTT assay. Results are expressed as mean±SD (n=4 per group). Statistical analysis was carried out using one-way ANOVA followed by Tukey test.

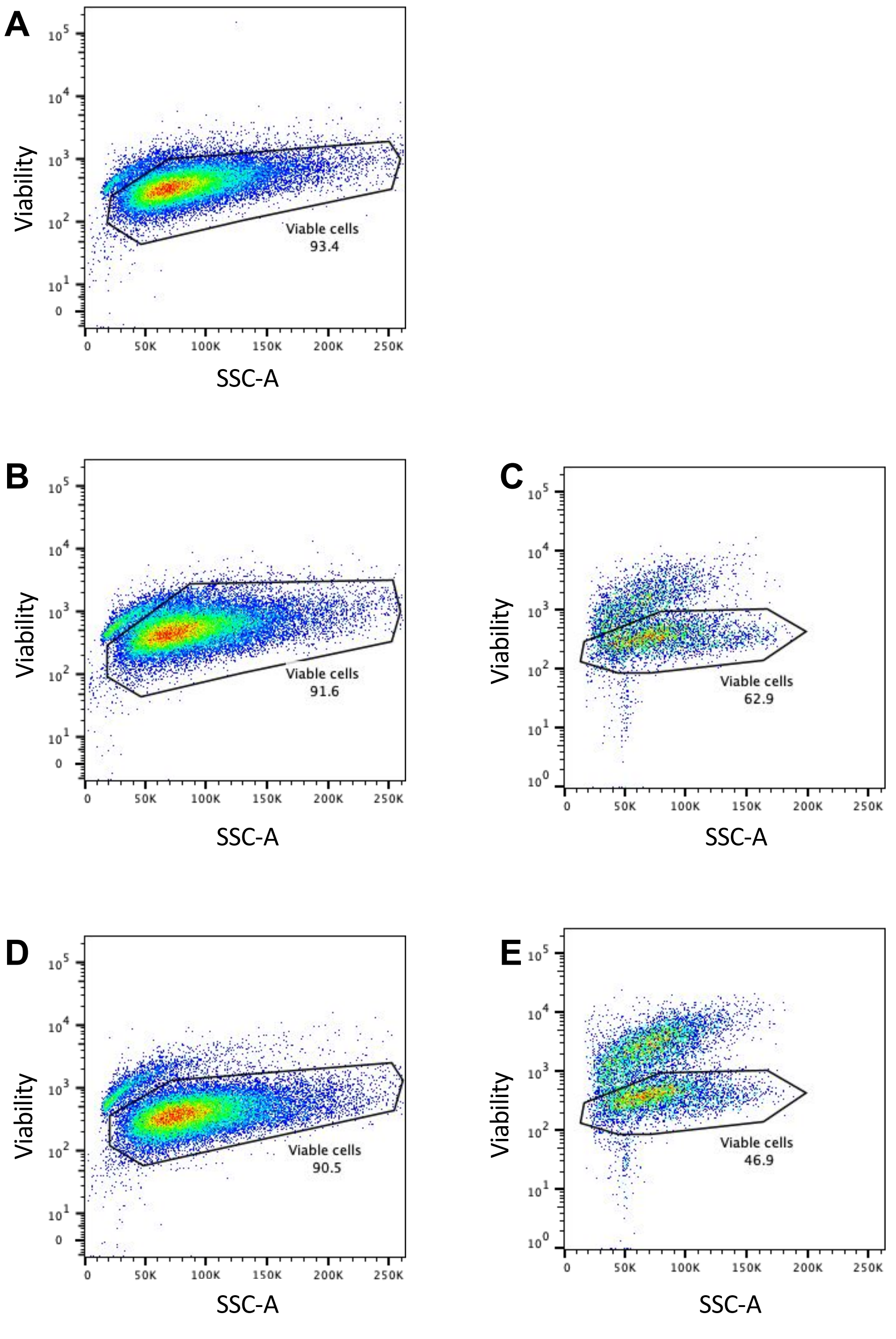

Figure S6. Cell viability is not decreased by nucleic acids but by doxorubicin. GL261 cells (n=3 per group) were treated with **(A)** media only, **(B)** siNEG LNP, **(C)** siNEG Dox LNP, **(D)** pIpC soluble and **(E)** pIpC-Dox LNP at 7.5µg/ ml nucleic acid and 0.18µM Doxorubicin, respectively. Nucleic acids, either soluble or encapsulated in LNPs did not result in cell death, whereas Doxorubicin did. Data was assessed by FlowJo Star v 10.8 software.

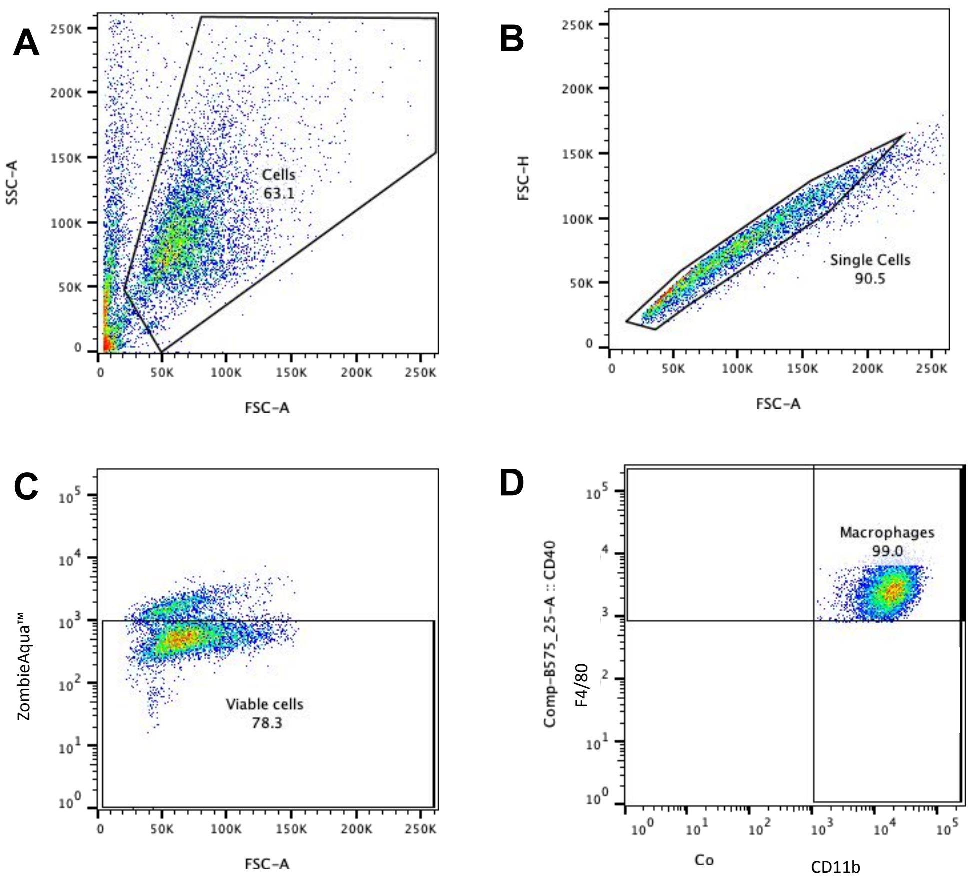

Figure S7. Gating strategy for *in vitro* flow cytometry analysis. GL261, J774 and bone marrow-derived cells (n=3 per group) were treated with different formulations prior to analysis via flow cytometry. . **(A)** Cells were distinguished from detritus by SSC-A vs. FSC-A gating strategy. **(B)** Single cells were identified via FSC-H vs. FSC-A gating strategy. **(C)** Viable cells were identified based on low intensity levels of ZombieAqua™ and high size (based on FSC-A). **(D)** The desired cell type was identified through expression levels of cell surface markers, for macrophages high levels F4/80 and CD11b. Data was assessed by FlowJo Star v 10.8 software.

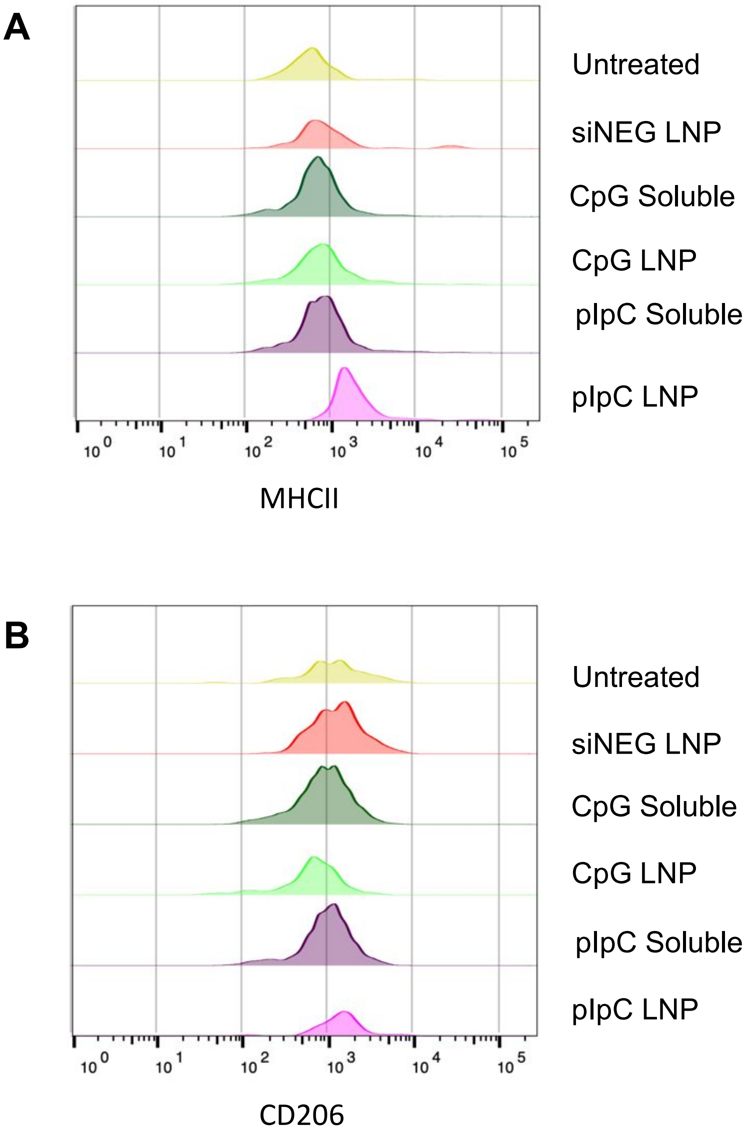

Figure S8. M2 to M1 macrophage reprogramming through pIpC LNPs. Cells isolated from mouse bone marrows were differentiated towards either an M0 phenotype by culturing in the presence of M-CSF. Following differentiation, cells were treated with immune adjuvants (CpG or pIpC) either in the soluble form or formulated in LNP (soluble or LNP) at 1µg/ml for 48h. LNPs formulated with siNEG (siNEG LNP) were used as a negative control. Cells were harvested and stained for ZombieAqua™, differentiation and activation markers before being acquired on FACSCelesta. **(A)** While the M1 phenotype marker showed an increase after treatment with the formulations, **(B)** the M2 phenotype marker CD206 did not change significantly. Data was assessed by FlowJo Star v 10.8 software.

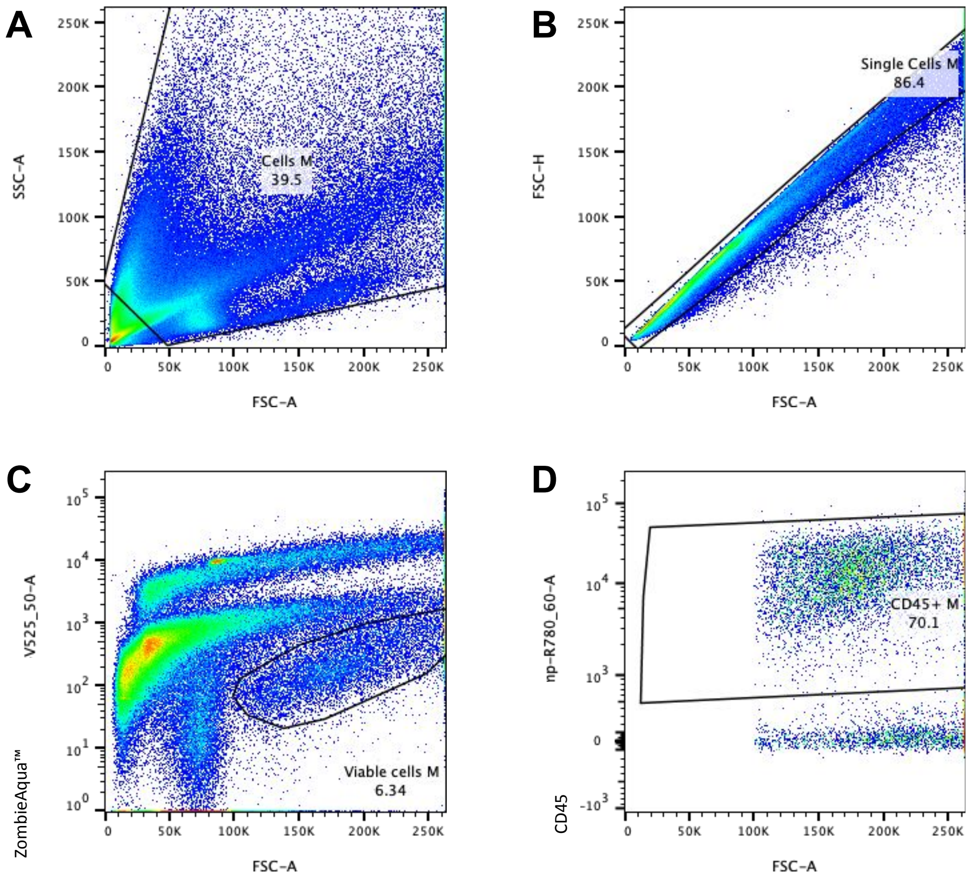

Figure S9. Gating strategy for *in vivo* flow cytometry analysis. GL261 brain tumour bearing (C57BL/6 mice, n=3 per group) were injected intracranially with LNP formulated TLR agonist (pIpC LNP, CpG LNP) or a pIpC LNP incorporating doxorubicin (pIpC LNP DOX) at a dose of 0.375µg/ g TLR agonist and 0.18µM Doxorubicin, on day 9 post tumour inoculation. As controls LNPs incorporating siNEG (siNEG LNP) or siNEG plus doxorubicin (siNEG DOX LNP) were used. A non-treated group (NT) served as a base line. Mice were sacrificed 3 days post treatment and a single cell suspension was isolated from extracted tumours. **(A)** Cells were distinguished from detritus by SSC-A vs. FSC-A gating strategy. **(B)** Single cells were identified via FSC-H vs. FSC-A gating strategy. **(C)** Viable cells were identified based on low intensity levels of ZombieAqua™ and high size (based on FSC-A). **(D)** Immune cells were identified based on high levels of CD45. Data was assessed by FlowJo Star v 10.8 software.

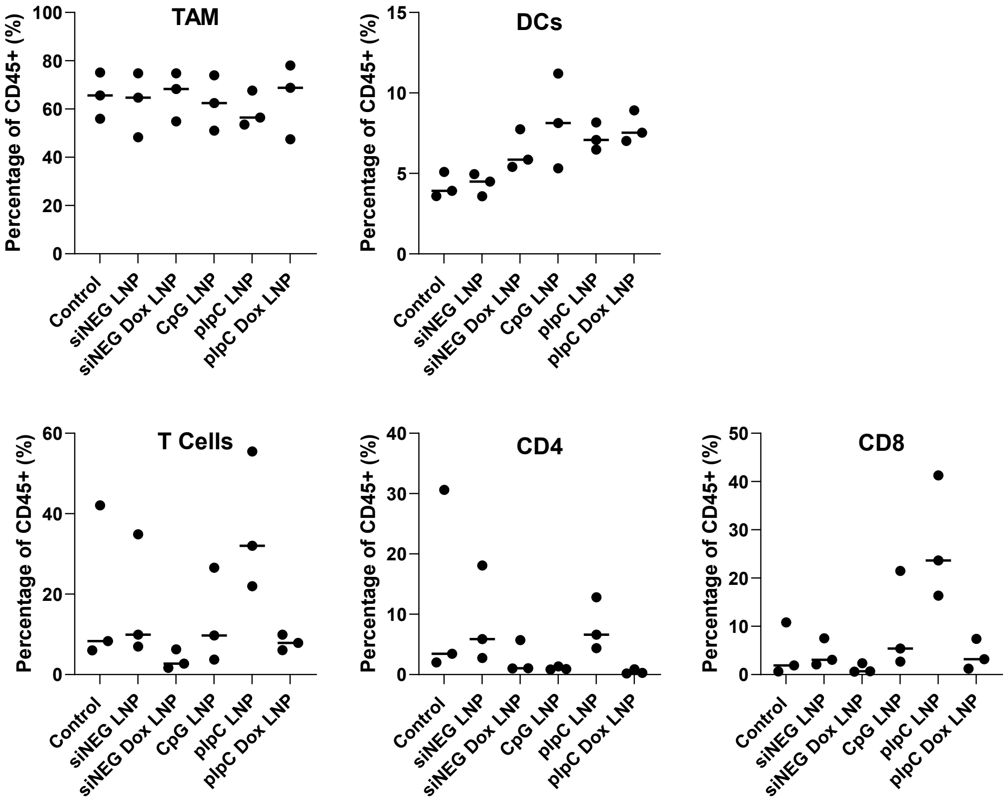

Figure S10. Ratio of tumor leukocyte populations following treatment with LNP formulations. Mice (C57BL/6, n=3 per group) were implanted i.c. with GL261 on day 0. At day 10, tumors were treated with LNP formulated nucleic acid (CpG or pIpC), with or without Doxorubicin (Dox). At day 13, mice were culled, tumors were excised and macerated to obtain a single cell suspension. Cells were stained Zombie Aqua viability dye, and a with a cocktail of monoclonal antibodies. Cells were acquired on a FACs Celesta. Data was analysed using Flowjo Software by first gating viable population (Zombie Aqua Low) then single cells (FSc Area vs FSc-Height). Next, the cell population of interest was assessed based on phenotypic markers as follows: Tumor associated macrophage (A) (TAM), CD45high, CD11bhigh; Dendritic cells (B) (DCs) CD45high, CD11chigh; T cells (C), CD45high,CD3high; CD4 T cells (D), CD45high,CD3high, CD4high; CD8 T cells (E), CD45high,CD3high, CD8high. In each case, data is presented as cell population of interest as a percentage of total leukocyte population (CD45+). Each point corresponds to an individual animal with the median of the group shown.

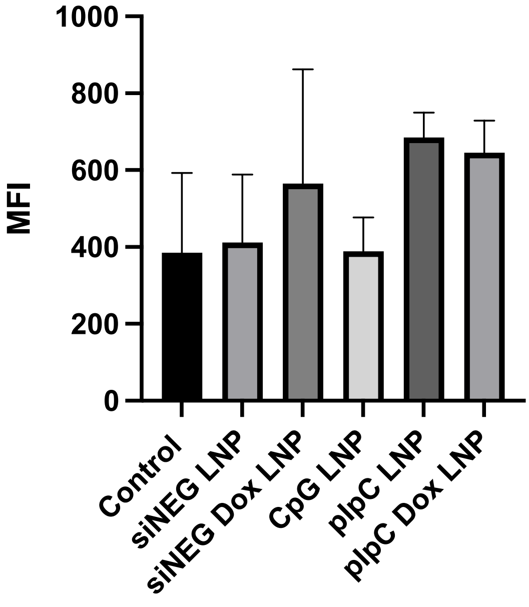

Figure S11. pIpC LNP and pIpC Dox LNP do not change activation status of CD4+ T cells *in vivo.* Animals bearing brain tumours were injected intracranially with nucleic acid only or in combination with Doxorubicin at a dosage of 7.5μg and 0.18μM respectively per mouse on day 9 post tumour inoculation. After 3 days CD4+ T cells were assessed for CD69 activation marker mean fluorescent intensity (MFI). FlowJo Star v 10.8 software was used to assess mean fluorescent intensity (MFI) on viable cells only. Data points represent mean and SD (n=3 per group). Statistical analysis was carried out using one-way ANOVA followed by Tukey test with significance of ****P<0.0001, ***P0.0002, **P0.0021 and *P0.0332.

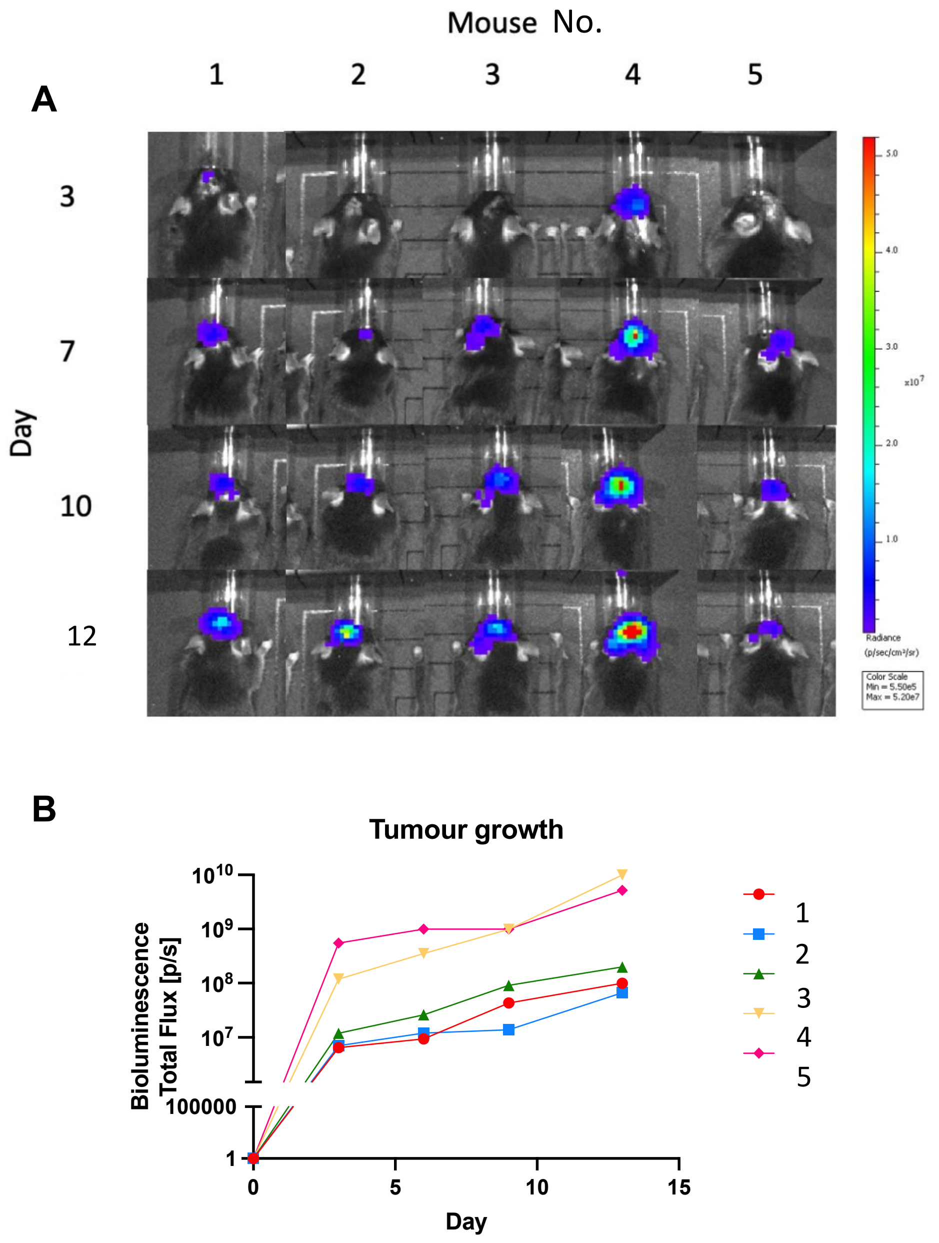

Figure S12. Tumour growth monitoring by bioluminescence imaging. Mice inoculated with GL261.luc cells on day 0 were imaged on days 3, 7, 10 and 13 (IVIS Lumina III, Perkin Elmer, UK). On day 13, animals were sacrificed for flow cytometric analysis of tumour microenvironment. **(A)** Whole head images. **(B)** Tumour growth curves displayed as total flux of bioluminescence signal [p/s].
